# Conserved mRNP remodeling mechanism of the TREX-2.1 complex

**DOI:** 10.1093/nar/gkag884

**Published:** 2026-09-12

**Authors:** Alexia E Angelos, Ryuta Asada, Bradley P Clarke, Pate S Hill, Lydia Li, Menghan Mei, Jalen L Smith, Ethan R Xie, Walter C Reter, Samuel J Smithee, Yihu Xie, Ben Montpetit, Yi Ren

**Affiliations:** Department of Biochemistry, Vanderbilt University School of Medicine, Nashville, TN 37232, United States; Department of Viticulture and Enology, University of California, Davis, CA 95616, United States; Department of Biochemistry, Vanderbilt University School of Medicine, Nashville, TN 37232, United States; Department of Biochemistry, Vanderbilt University School of Medicine, Nashville, TN 37232, United States; Department of Biochemistry, Vanderbilt University School of Medicine, Nashville, TN 37232, United States; Department of Biochemistry, Vanderbilt University School of Medicine, Nashville, TN 37232, United States; Department of Biochemistry, Vanderbilt University School of Medicine, Nashville, TN 37232, United States; Aspirnaut Program, Vanderbilt University Medical Center, Nashville, TN 37232, United States; Department of Biochemistry, Vanderbilt University School of Medicine, Nashville, TN 37232, United States; Department of Biochemistry, Vanderbilt University School of Medicine, Nashville, TN 37232, United States; Department of Biochemistry, Vanderbilt University School of Medicine, Nashville, TN 37232, United States; Department of Biochemistry, Vanderbilt University School of Medicine, Nashville, TN 37232, United States; Department of Viticulture and Enology, University of California, Davis, CA 95616, United States; Department of Biochemistry, Vanderbilt University School of Medicine, Nashville, TN 37232, United States; Center for Structural Biology, Vanderbilt University, Nashville, TN 37232, United States

## Abstract

Processing, packaging, and nuclear export of messenger ribonucleoprotein particles (mRNPs) are critical for eukaryotic gene expression, with the DEAD-box ATPase DDX39B (yeast Sub2) playing a central role in mRNP processing and remodeling. Our recent studies identified human TREX-2 (GANP•PCID2•DSS1), yeast TREX-2 (Sac3•Thp1•Sem1), and a related human TREX-2.1 complex (LENG8•PCID2•DSS1) as key regulators of DDX39B/Sub2. Here, we characterize the yeast TREX-2.1 (scTREX-2.1) complex, composed of Thp3, Csn12, and Sem1. We show that the scTREX-2.1 complex directly interacts with Sub2 and co-occupies a fraction of CBC-containing mRNPs with Sub2. Using cryo-electron microscopy , we determined the structure of scTREX-2.1 bound to Sub2, revealing a conserved “trigger loop” mechanism by which scTREX-2.1 regulates Sub2 activity. Functional assays show that disruption of scTREX-2.1 leads to the accumulation of intron-containing pre-mRNAs. These findings uncover a conserved mechanism from yeast to humans by which TREX-2 and TREX-2.1 complexes regulate Sub2/DDX39B during nuclear mRNP maturation, providing insights into the coordination of mRNP remodeling and processing prior to nuclear export.

## Graphical Abstract

**Graphical Abstract:**
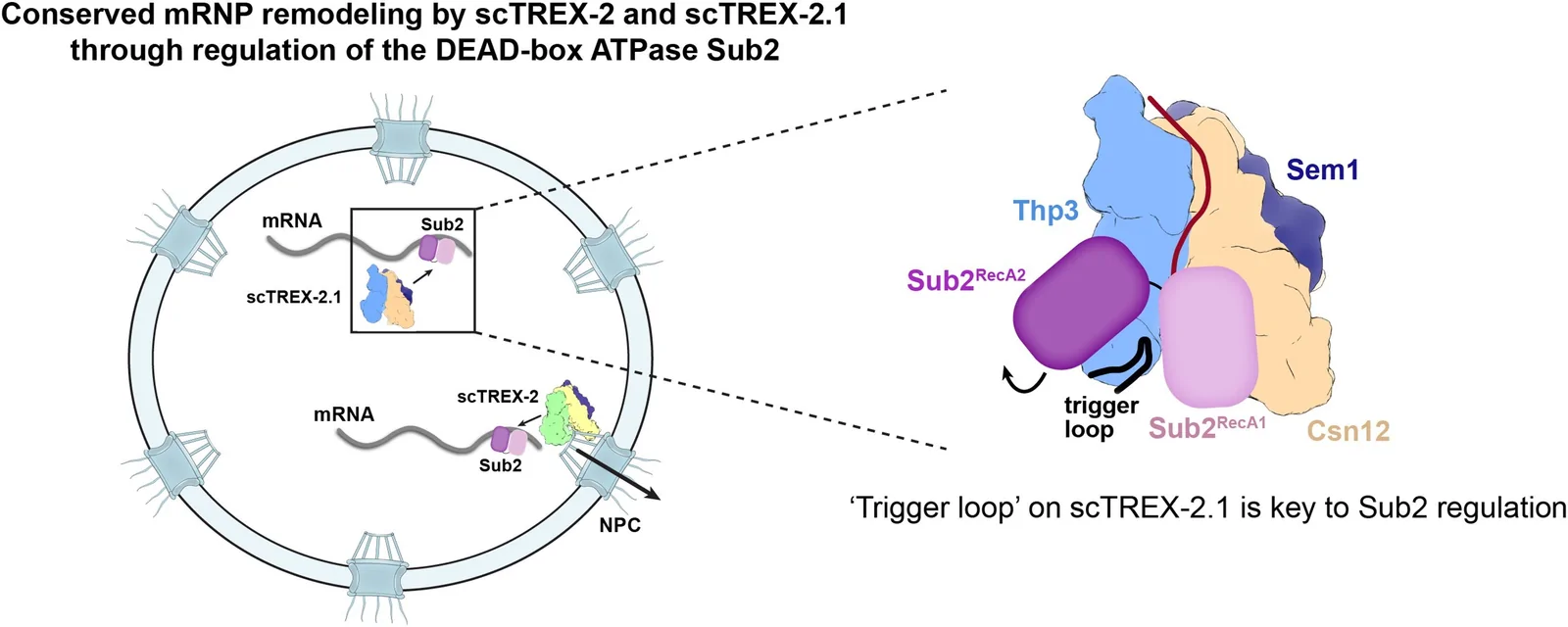

## Introduction

Eukaryotic mRNAs are packaged with RNA-binding proteins as ribonucleoprotein particles (mRNPs) [1–6]. In the nucleus, proper assembly and remodeling of mRNPs are critical for the processing and nuclear export of transcripts through the nuclear pore complex (NPC) [7–14]. A key component of nuclear mRNPs is the TREX (TRanscription-EXport) complex, which consists of the THO complex, the DEAD-box ATPase DDX39B (also known as UAP56; yeast Sub2), and ALYREF (yeast Yra1) [15–26]. TREX and related factors associate with actively transcribed genes [25, 27, 28], engage multiple factors involved in mRNA processing [29–35], mediate mRNP compaction [7, 8, 36], and facilitate recruitment of the principal mRNA export receptor NXF1•NXT1 (yeast Mex67•Mtr2) to form export-competent mRNPs [2, 37]. Through direct interaction with NXF1•NXT1, the nuclear basket-associated TREX-2 complex functions at a late step of mRNP export, immediately before entry into the NPC channel [38–49]. The integrated functions of TREX and TREX-2 are further revealed by recent studies identifying TREX-2 as a conserved regulator of Sub2/DDX39B from yeast to humans during mRNP remodeling [50–53].

As a core component of TREX and the factor that functionally connects TREX to TREX-2, the activity of the DEAD-box ATPase Sub2/DDX39B is central to nuclear mRNP packaging and remodeling. The THO complex associates with Sub2 on actively transcribed genes [24] and can bind Sub2 in a half-open conformation, as demonstrated by a crystal structure of the THO•Sub2 complex [54] and later corroborated by cryo-electron microscopy (cryo-EM) structures [55–58]. When engaged with mRNPs, Sub2 adopts a closed conformation and can form higher-order complexes to facilitate mRNP assembly, as demonstrated by crystal structures of Sub2•Yra1•RNA and DDX39B•Tho1•RNA [36, 54]. Sub2/DDX39B does not accompany the mRNP to the cytoplasm [59] and is released from mRNPs prior to export, with the TREX-2 complex utilizing a conserved “trigger loop” to stimulate Sub2 ATPase activity and its release from the mRNA [50]. The molecular basis of Sub2 recognition by TREX-2 is demonstrated by a cryo-EM structure of the yeast TREX-2•Sub2 complex [50]. Furthermore, this mechanism is conserved, as revealed by cryo-EM structures of the human TREX-2•DDX39B complex [51–53]. These findings support a model in which a series of interactions with regulators, e.g. THO and TREX-2, facilitates the binding and release of Sub2/DDX39B from the mRNP during nuclear mRNP processing and remodeling to ultimately promote mRNP export through an NPC.

Our recent work identified a TREX-2-related complex, TREX-2.1, in humans [51]. TREX-2.1 is composed of LENG8, PCID2, and DSS1, which, like TREX-2, uses a conserved trigger loop to regulate the mRNP remodeling activity of human DDX39B, as shown by a cryo-EM structure of the TREX-2.1•DDX39B complex [51]. In this case, TREX-2.1 influences the nucleocytoplasmic ratio of a subset of mRNAs with high GC content, which are distinct from those regulated by TREX-2 [51]. Similarly, in yeast, the Sem1 subunit of TREX-2, together with Thp3 and Csn12, forms a TREX-2-like complex [60–62], which we refer to as the scTREX-2.1 complex. Yeast TREX-2.1 is suggested to be the ortholog of the human TREX-2.1 complex based on sequence homology between Thp3 in scTREX-2.1 and LENG8 in hsTREX-2.1 [63].

Unlike NPC-localized TREX-2, NPC targeting sequences are absent in TREX-2.1. Consistently, both yeast and human TREX-2.1 are nucleoplasmic [51, 64], suggesting that these complexes may function at earlier stages of nuclear mRNP biogenesis. scTREX-2.1 has been shown to be recruited to actively transcribed genes and affect transcription elongation [61]. The Csn12 subunit exhibits genetic interaction profiles similar to components of the spliceosome, such as Isy1, with deletion of either *CSN12* or *THP3* causing pre-mRNA accumulation in intron-containing genes [60], suggesting a functional link between scTREX-2.1 and splicing. Recent evidence suggests that both yeast and human TREX-2.1 couple splicing factors with the nuclear exosome mRNP decay pathway [65–67], and human TREX-2.1 was also shown to modulate alternative mRNA processing [68]. These data provide evidence of a conserved mechanism between yeast and human TREX-2.1; however, how scTREX-2.1 functions at a molecular level remains largely unexplored.

Here, we show that the scTREX-2.1 complex directly regulates Sub2 activity *in vitro* and is found with Sub2 in mRNPs formed *in vivo*. A cryo-EM structure of the scTREX-2.1•Sub2 complex further reveals the underlying molecular basis of their interaction and demonstrates a conserved trigger loop mechanism by which TREX-2 and TREX-2.1 regulate Sub2/DDX39B during nuclear mRNP remodeling.

## Materials and methods

### Reagents

Reagents used in this study are listed in Supplementary Table S1.

### Biological resources

All plasmids and oligos used in this study are listed in Supplementary Tables S2 and S3. Yeast strains used in this study are listed in Supplementary Table S4.

### Protein expression and purification

The scTREX-2.1 complex was obtained by co-expressing the plasmids encoding GST-Thp3 (wild type or mutant) and the His-Csn12•Sem1 complex in *Escherichia coli* Rosetta cells (Sigma–Aldrich). Protein expression in Rosetta cells was induced by 0.5 mM Isopropyl β-D-thiogalactoside (IPTG) at 20°C overnight. Cells were lysed in a lysis buffer containing 50 mM Tris (pH 8.0), 300 mM NaCl, 20 mM Imidazole, 0.5 mM TCEP, 0.1 mM AEBSF, and 2 mg/l aprotinin. The scTREX-2.1 complex was subjected to tandem affinity purification, first with Ni Sepharose 6 fast flow resin (Cytiva) and subsequently with Glutathione Sepharose 4B resin (Cytiva) to obtain a stoichiometric complex. Next, expression tags were cleaved with GST-TEV at 4°C overnight. Digested protein was purified on a Source 15S column (Cytiva) using a linear gradient of NaCl in 10 mM Tris (pH 8.0) and 0.5 mM TCEP. The proteins were further purified on a Superdex 200 column (Cytiva) equilibrated with 10 mM Tris (pH 8.0), 200 mM NaCl, and 0.5 mM TCEP.

Sub2 and GST-tagged Sub2 were purified as previously described [50]. For the expression of *p*-benzoyl-l-phenylalanine (*p*Bpa)-incorporated Sub2, the Sub2 expression plasmid with the amber codon at residues 68, 78, or 125 was co-transformed with an aminoacyl-tRNA synthetase/tRNA pair encoded by the plasmid pEVOL-pBpF (Addgene, 31190) in *E. coli* BL21 (DE3) (NEB). Arabinose was added to the cell culture at a final concentration of 0.02% (w/v) when the optical density (OD) at 600 nm reached 0.3 to induce expression of the aminoacyl-tRNA synthetase/tRNA pair. Cells were spun down when the OD at 600 nm reached 0.6 and resuspended in a volume of media five-fold less than the initial culture volume in the presence of 1 mM *p*Bpa (AEchem) and 0.02% (w/v) arabinose. Cells were incubated at 16°C for 1 h, and protein expression was then induced with 0.5 mM IPTG at 16°C for 16 h. The *p*Bpa-incorporated Sub2 proteins were pulled down using Glutathione Sepharose 4B resin, followed by GST tag cleavage by GST-TEV. Protein was then purified on a Source 15Q column using a linear gradient of NaCl in 10 mM Tris (pH 8.0) and 0.5 mM TCEP and was further purified on a Superdex 200 column equilibrated with 10 mM Tris (pH 8.0), 300 mM NaCl, and 0.5 mM TCEP.

### mRNP-SiMPull

mRNP-SiMPull assay (stoichiometry analysis of Thp3 and co-localization analysis of Thp3-Sub2, Sub2-Hpr1, and Hpr1-Thp3) was conducted as described previously [8] using yeast strains listed in Supplementary Table S4. For Thp3-mNG photobleaching step analysis, nuclear mRNPs were isolated by on-bead pulldown through Cbp80-PrA using rabbit IgG conjugated magnetic beads, and the eluted mRNPs captured on a glass coverslip using anti-mNG antibody (Chromotek, 32F6). For co-localization analysis, nuclear mRNPs were purified by Cbp80 pulldown then captured on the coverslip through Cbp20-3V5 (for Thp3-Sub2 and Hpr1-Sub2 co-localization analysis) using anti-V5 antibody (Invitrogen, R960-25) or Thp3-SNAPf-3HA or Hpr1-mNG (for Thp3-Hpr1 co-localization analysis) using anti-HA (Sigma, H3663) or anti-mNG antibody, respectively.

### Photo-crosslinking with *p*Bpa-incorporated Sub2

For mass spectrometry experiments, 5 µM of scTREX-2.1 was incubated with 5 µM of Sub2^L68^*^p^*^Bpa^, Sub2^I78^*^p^*^Bpa^, or Sub2^D125^*^p^*^Bpa^ on ice for 30 min in buffer containing 10 mM Tris (pH 8.0), 100 mM NaCl, and 0.5 mM TCEP. Protein mixtures were irradiated at 365 nm (Spectrolinker) on ice in a 96-well plate. Reactions were irradiated for 30 min in 5-min intervals with 1-min breaks for cooling on ice between crosslinking.

To generate crosslinked scTREX-2.1•Sub2^D125^*^p^*^Bpa^ complex for cryo-EM studies, 6 µM of scTREX-2.1 was incubated with 15 µM of Sub2^D125^*^p^*^Bpa^ on ice for 30 min in a buffer containing 10 mM Tris (pH 8.0), 75 mM NaCl, 1 mM ADP, 1 mM MgCl_2_, and 0.5 mM TCEP. The protein mixture was irradiated at 365 nm on ice in a 96-well plate for 40 min in 3-min intervals with 2-min breaks for cooling on ice between crosslinking.

### Mass spectrometry

Photo-crosslinked samples for each Sub2*^p^*^Bpa^ construct tested were resolved on an SDS–PAGE (sodium dodecyl sulfate–polyacrylamide gel electrophoresis) gel. The bands corresponding to crosslinked products were excised and subjected to in-gel digestion using trypsin for Sub2^L68^*^p^*^Bpa^ crosslinked products and using trypsin and Glu-C for Sub2^I78^*^p^*^Bpa^ and Sub2^D125^*^p^*^Bpa^ crosslinked products. The resulting peptides were analyzed by Liquid chromatography-tandem mass spectrometry (LC–MS/MS) utilizing an Orbitrap Exploris 240 mass spectrometer (Thermo). Resulting MS/MS spectra were analyzed using the pLink3 software suite using default parameters, with the exception that the minimum peptide length was set to 4 to accommodate shorter *p*Bpa-containing peptides [69]. A 5% false discovery rate cutoff was used to determine interprotein crosslinks. A summary of the Sub2^L68^*^p^*^Bpa^•scTREX-2.1 and Sub2^I78^*^p^*^Bpa^•scTREX-2.1 crosslinks is shown in Supplementary Tables S5 and S6.

### Cryo-EM data collection, image processing, and model building

The photo-crosslinked scTREX-2.1•Sub2^D125^*^p^*^Bpa^ sample, in a buffer containing 10 mM Tris (pH 8.0), 75 mM NaCl, 1 mM ADP, 1 mM MgCl_2_, and 0.5 mM TCEP, was deposited on glow-discharged UltrAuFoil R 1.2/1.3 grids (Quantifoil). Grids were blotted for 3 s with a blotting force of 6 at room temperature and 100% humidity and plunged into liquid ethane using a Vitrobot Mark IV (Thermo). The data were collected with a Titan Krios G4 (Thermo Fisher) equipped with a K3 direct electron detector (Thermo Fisher). Movies were collected with EPU at a magnification of 105 000× with a defocus range from −0.8 to −2.0 μm. Cryo-EM data collection parameters can be found in Supplementary Table S7.

Cryo-EM data were processed with cryoSPARC [70]. Movies were gain-normalized, aligned, and dose-weighted using patch motion correction, followed by patch CTF estimation. After manual curation, 10 497 micrographs were kept for further processing. A subset of 1000 micrographs was used during pilot processing to obtain a set of clean particles to generate a Topaz model. For the entire dataset, 11.4 million particles were picked with the Topaz model. *Ab-initio* reconstruction and multiple rounds of heterogeneous refinement yielded two maps that both feature densities for scTREX-2.1, and one of the maps additionally shows densities for Sub2. The 2.6 million particles that correspond to the scTREX-2.1•Sub2 complex were subjected to multiple rounds of heterogeneous refinement, 3D classification, reference-based motion correction, and nonuniform refinement. A final set of 57 575 particles generated a scTREX-2.1•Sub2 reconstruction at 3.72 Å resolution.

For model building, the scTREX-2.1 (PDB ID 7EWM) and Sub2 (PDB ID 5SUP) crystal structures were docked into the cryo-EM map as an initial model. The model was manually adjusted in Coot and refined in Phenix with real-space refinement [71, 72]. The final model contains the trimeric scTREX-2.1 complex and the N-terminal motif (NTM) and RecA1 domain of Sub2.

### ATPase assay

ATPase activity was analyzed using an NADH enzyme-coupled absorbance assay. ATPase reactions were prepared with 1.5 μM of indicated proteins (Sub2, scTREX-2.1, or scTREX-2.1-mut) in 10 mM Tris (pH 8.0), 120 mM NaCl, 2 mM MgCl_2_, 0.5 mM TCEP, 0.125 mg/ml poly(A) RNA (Cytiva), 1 mM ATP, 5 mM phosphoenolpyruvate, 1.2 mM (for reactions with both Sub2 and scTREX-2.1) or 0.1 mM NADH (other reactions), and 2% (v/v) pyruvate kinase/lactate dehydrogenase (Sigma). UV absorbance at 340 nm was monitored by a BioTek Synergy HTX microplate reader at 37°C. Reaction rates were calculated in GraphPad Prism 10 from the slopes of the linear phase showing the decrease in NADH absorbance as a function of time.

### GST pulldown

Two micromolars of GST or GST-tagged Sub2 was mixed with purified scTREX-2.1 in a binding buffer containing 20 mM Tris (pH 8.0), 150 mM NaCl, and 0.5 mM TCEP. Samples were incubated with Glutathione Sepharose 4B resin on ice for 30 min, with gentle tapping every 3–5 min. Resins were washed with 600 µl binding buffer three times. Proteins remaining on the resin were eluted with buffer containing 20 mM Tris (pH 8.0), 150 mM NaCl, 0.5 mM TCEP, and 20 mM reduced glutathione and resolved on an SDS–PAGE gel and stained with Instant Blue.

### Csn12-GFP degradation in yeast with the AlissAID system

The CSN12-GFP yeast strain (Supplementary Table S4) was obtained from the Yeast GFP Clone Collection (Invitrogen). Plasmid BYP10392 [NBRP, pAlissAID anti-GFP_316 (URA3 marker)] was transformed into the CSN12-GFP strain using the lithium acetate method.

The BYP10392-transformed CSN12-GFP yeast strain was streaked from a glycerol stock onto an SD-URA (DOB plus CSM-URA, Sunrise Science Products) plate and grown at 30°C. A single colony was used to start a liquid culture in SD-URA at 30°C. Once the culture reached an OD_600_ of ∼0.4, 5-Adamantyl-IAA (5-Ad-IAA) was added to the culture at a final concentration of 5 µM. Approximately 3 × 10^7^ cells were collected at each timepoint of 0, 30, 60, and 120 min and spun down. Pellets were washed once with sterile water and stored at −80°C. Experiments were repeated with three different BYP10392 transformants.

### Western blot

Proteins were extracted from frozen pellets collected during the AlissAID protein degradation time course. Twenty percent trichloroacetic acid (TCA) and glass beads were added to the pellets, and cells were disrupted using a bead beater. Lysates were washed with 5% TCA and pelleted. The pellet was resuspended with 1× SDS loading buffer, and proteins were resolved on a 12% SDS–PAGE gel. Proteins were transferred to a 0.45-µm nitrocellulose membrane using the Trans-Blot Turbo Transfer System (Bio-Rad). Csn12-GFP was detected using mouse anti-GFP antibody (Sigma, 11814460001) at a dilution of 1:1000. PGK1 was detected using mouse anti-PGK1 (Abcam, ab113687) at a dilution of 1:10000. Secondary antibodies were HRP-labeled horse anti-mouse IgG (Cell Signaling, 7076) and were used at 1:2000 (GFP blots) or 1:6000 (PGK1 blots).

### RNA extraction and RT-qPCR

RNA was extracted from frozen pellets collected during the AlissAID protein degradation time course. Pellets were resuspended in TRIzol (Invitrogen) and mixed with glass beads. Cells were then disrupted using a bead beater. Nucleic acids were extracted using chloroform, as per the TRIzol manufacturer’s protocol. A second chloroform extraction was performed with the aqueous phase of the first extraction. RNA was then precipitated with isopropanol, washed with 75% ethanol, and dissolved in RNase-free water. RNA quality was assessed via gel electrophoresis. DNase digestion was performed with RQ1 RNase-free DNase (Promega) as per the manufacturer’s protocol. The enzyme was heat inactivated. To generate complementary DNA (cDNA), 1 µg of RNA was used with SuperScript III First-Strand cDNA Synthesis System (Thermo) as per the manufacturer’s protocol using random hexamer primers.

qPCR was performed using the Fast SYBR Green Master Mix (Thermo) and a QuantStudio 3 Real-Time PCR System (Thermo). Experiments were performed in technical triplicate with three different BYP10392 transformants. Intron-containing transcripts were detected using primers specific to intron–exon junctions. Relative intron levels were calculated using the ΔΔCt method with the intronless gene ALG9 as an internal control. The primers used for reverse transcription quantitative PCR (RT-qPCR) are listed in Supplementary Table S3.

### Fluorescence microscopy

The BYP10392-transformed CSN12-GFP yeast strain was grown and treated with 5-Ad-IAA as described in the AlissAID section. At timepoints 0′ and 120′, ∼1 OD_600_ unit of yeast cells was collected by centrifugation. Yeast pellets were washed once with sterile water, spun down, and resuspended in 30 µl of sterile water. Three microliters of cell suspension was spread on Concanavalin-A-coated glass coverslips. Coverslips were then mounted onto glass slides. Cells were allowed to settle for 5 min before immediate imaging. Live cells were imaged on a Nikon Eclipse Ti widefield fluorescence microscope equipped with a DS-Qi2 camera, using a 100×/1.45 NA oil immersion objective. GFP signal was obtained using a 470/30 nm excitation filter and a 520/40 nm emission filter. Imaging was repeated with three different BYP10392 transformants. Images were analyzed in Fiji.

### Fluorescent *in situ* hybridization

Poly(A) RNA fluorescent *in situ* hybridization (FISH) is conducted by following smFISH protocol as described previously [8] using custom designed Cy3-labeled LNA oligo dT (25 Ts) probe (Qiagen) with the final concentration 50 nM during hybridization. Images are acquired by confocal microscopy (Leica 63 × 1.4 NA objective on a DMi8 stand connected to an Andor Dragonfly microscope and an iXon Ultra 888 EMCCD camera).

## Results

### The scTREX-2.1 complex directly interacts with the DEAD-box ATPase Sub2

**Figure 1.**
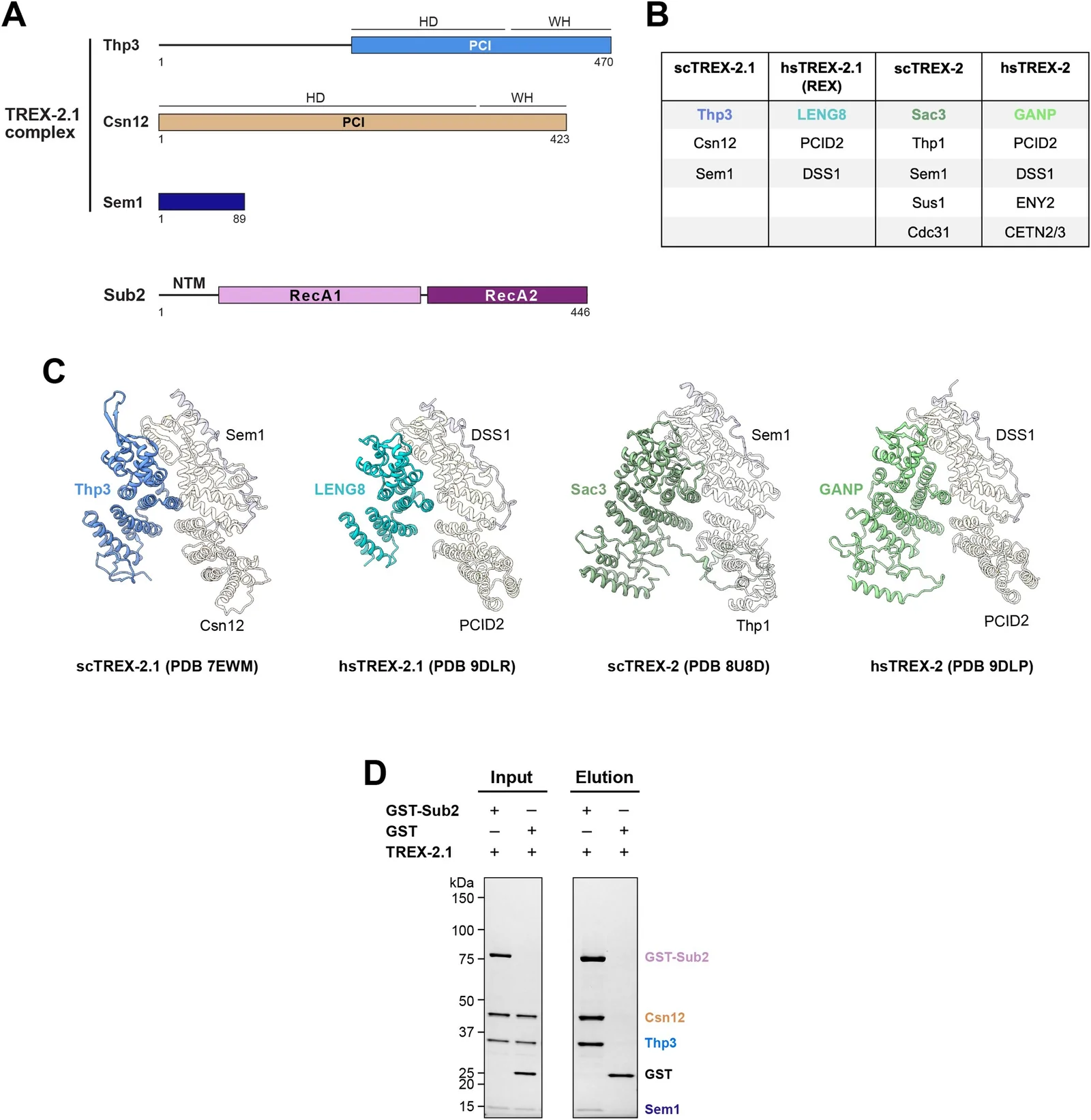
The scTREX-2.1 complex directly interacts with Sub2. (**A**) Schematic representations of the scTREX-2.1 (Thp3•Csn12•Sem1) complex and Sub2. (**B**) Composition of TREX-2 and TREX-2.1 complexes in yeast and humans. (**C**) Structural alignment of the yeast TREX-2.1 complex (PDB ID 7EWM), the human TREX-2.1 complex (PDB ID 9DLR), the yeast TREX-2 complex (PDB ID 8U8D), and the human TREX-2 complex (PDB ID 9DLP). (**D**) scTREX-2.1 directly interacts with Sub2. *In vitro* GST pulldown was performed with purified GST-Sub2 and the scTREX-2.1 complex.

The scTREX-2.1 and scTREX-2 complexes share only one component, the small 89-residue subunit Sem1 (Fig. 1A and B). The other two subunits of the scTREX-2.1 complex, Thp3 and Csn12, show moderate homology to the Sac3 and Thp1 subunits of scTREX-2, respectively. Despite their distinct protein compositions, the “V” shaped architecture of scTREX-2.1 resembles the middle region of the scTREX-2 complex, consisting of Sac3, Thp1, and Sem1 (Fig. 1C). This architecture is also conserved in the human TREX-2 and TREX-2.1 complexes (Fig. 1B and C) [51, 65–68]. For each of these complexes, the two larger subunits feature a PCI fold [73], which is composed of an N-terminal helical domain (HD) and a C-terminal winged-helix domain (WH) (Supplementary Fig. S1A). In scTREX-2.1, Thp3 and Csn12 interact through their WH domains, forming the “V” shaped architecture, and Sem1 binds to Csn12 (Fig. 1C and Supplementary Fig. S1B). Structure-based alignment shows that Thp3 resembles LENG8 of the human TREX-2.1 complex more closely (30% sequence identity; RMSD 1.8 Å) than Sac3 of the scTREX-2 complex (20% sequence identity; RMSD 2.4 Å) (Fig. 1C) [74], suggesting scTREX-2.1 is the ortholog of the human TREX-2.1 complex.

We previously showed that scTREX-2 as well as human TREX-2 and TREX-2.1 directly interact with Sub2/DDX39B [50, 51], but to date, it is not known whether Sub2 binding is also conserved in scTREX-2.1. Thus, we tested whether scTREX-2.1 directly interacts with Sub2 using a recombinant scTREX-2.1 complex containing Thp3 (residues 140–470), full-length Csn12, and full-length Sem1 through co-expression in *E. coli*. The N-terminal region of Thp3 is predicted to be disordered and was removed to obtain a soluble scTREX-2.1 complex. Full-length Sub2 was used in this study; Sub2 contains an unstructured NTM and two RecA domains connected by a short linker. Using purified recombinant proteins, GST-Sub2 was able to pull down a stoichiometric amount of the scTREX-2.1 complex (Fig. 1D). Although the two Sub2-interacting subunits in the TREX-2 complex, Sac3 and Thp1, are replaced by Thp3 and Csn12 in scTREX-2.1, respectively, scTREX-2.1 still directly interacts with Sub2. These results demonstrate the plasticity of Sub2 in engaging different binding partners, which may differentially regulate the ATPase activity of Sub2.

### scTREX-2.1 and Sub2 co-occupy nuclear mRNPs

**Figure 2.**
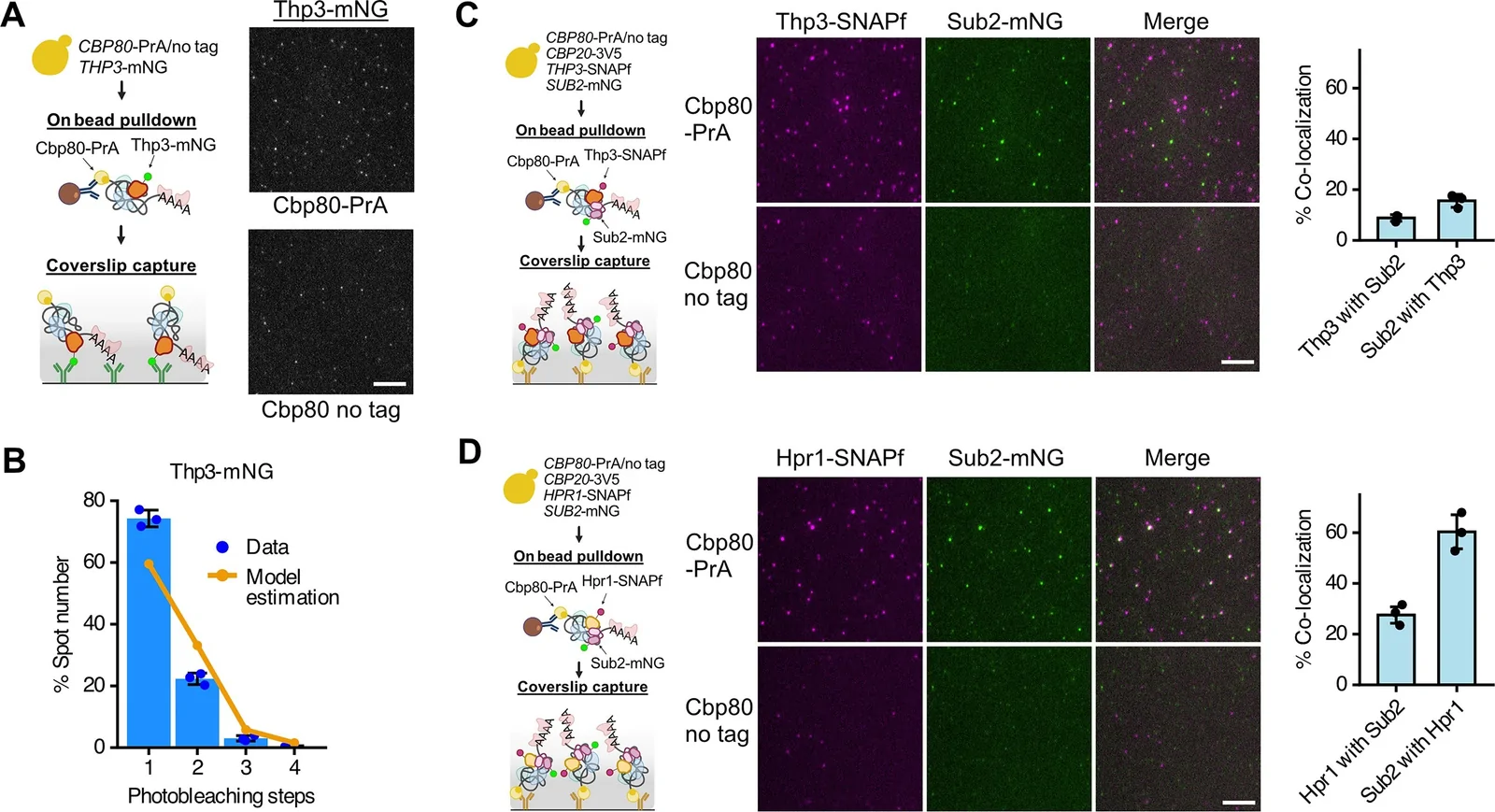
scTREX-2.1 and Sub2 co-occupy CBC-containing mRNPs. (**A**) Cartoon depicts mRNP isolation procedure to capture and determine the stoichiometry of the scTREX-2.1 component, Thp3, in Cbp80-bound mRNPs using TIRF microscopy. Representative TIRF images of Thp3-mNG in the pulldown. Scale bar, 5 µm. (**B**) Graph shows stoichiometry distribution determined by photobleaching step analysis. Blue bars show mean data with error bars displaying standard deviation. Individual biological replicates are shown as blue dots. Orange line displays the expected stoichiometry distribution following correction for fluorescent reporter activity using finite mixture modeling as described in Asada *et al.* [8]. (**C, D**) Cartoons depict mRNP isolation procedure to capture and determine co-localization of Thp3-SNAPf and Hpr1-SNAPf with Sub2-mNG in CBC-bound mRNPs by TIRF microscopy. Representative TIRF images of Thp3-SNAPf or Hpr1-SNAPf with Sub2-mNG. Scale bar, 5 µm. Graphs show percent co-localization frequency with error bars showing standard deviation and replicate data as black dots.

To verify that scTREX-2.1 occupies nuclear mRNPs *in vivo*, we examined scTREX-2.1 association with mRNPs isolated from yeast using the single-molecule imaging technique mRNP-SiMPull [8]. Yeast nuclear mRNPs were isolated through a two-step pulldown that first captures protein A (PrA)-tagged Cbp80 on bead, a component of the nuclear cap-binding complex (CBC), and then fluorescently tagged scTREX-2.1 in the second step on a TIRF imaging slide (Fig. 2A). Thp3-associated complexes were significantly enriched compared to the control that lacks a tag on Cbp80, indicating that scTREX-2.1 is indeed found in a fraction of CBC-containing mRNPs. From these data, scTREX-2.1 stoichiometry was measured by photobleaching step analysis and found to be one to two copies per mRNP (Fig. 2B).

Next, we examined whether scTREX-2.1 and Sub2 can co-occupy the same individual mRNP via co-localization analysis of Thp3 and Sub2 in comparison to another Sub2 interaction partner Hpr1, a component of the THO complex. Due to high background when assaying Sub2-mNG [8], we employed Cbp20-3V5 (CBC component) to capture CBC-bound mRNPs on the glass surface after Cbp80-PrA bead capture to perform co-localization analysis with Sub2 (Fig. 2C and D). In the case of Thp3 co-localization analysis with Hpr1, target proteins were directly captured on the glass surface using an antibody to a fusion tag on Thp3 or Hpr1 (Supplementary Fig. S2A). Using these approaches, ∼9% of the total Thp3 spots co-localized with Sub2 (Fig. 2C). These data indicate that scTREX-2.1 can co-occupy an mRNP with Sub2; however, the majority of scTREX-2.1-bound mRNPs lack Sub2. We also assessed the reverse co-localization frequency (i.e. Thp3 co-localization on total Sub2 spots) and found that ∼16% Sub2 spots contain Thp3 (Fig. 2C). In comparison, ∼28% of Hpr1 spots were co-localized with Sub2 and ∼60% of Sub2 spots co-localized with Hpr1 (Fig. 2D). These co-localization frequencies of Sub2 with Hpr1 and Thp3 are consistent with Sub2 dynamically binding to different types of mRNPs, more commonly with the THO complex, but also with TREX-2.1. Finally, we examined co-localization between Thp3 and Hpr1 to address whether both the THO complex and TREX-2.1 can be present in a single mRNP (Supplementary Fig. S2B and C). In this case, ∼19% of Thp3 co-localized with Hpr1, while ∼14% of Hpr1 spots co-localized with Thp3 (Supplementary Fig. S2D). These data suggest the existence of a heterogenous mixture of mRNPs across nuclear contexts (e.g. mRNP processing and/or quality control), including the formation of mRNPs with both TREX and TREX-2.1 engaged with the same mRNA.

### Probing scTREX-2.1 interactions with Sub2 using photoactivatable Sub2^*p*Bpa^

**Figure 3.**
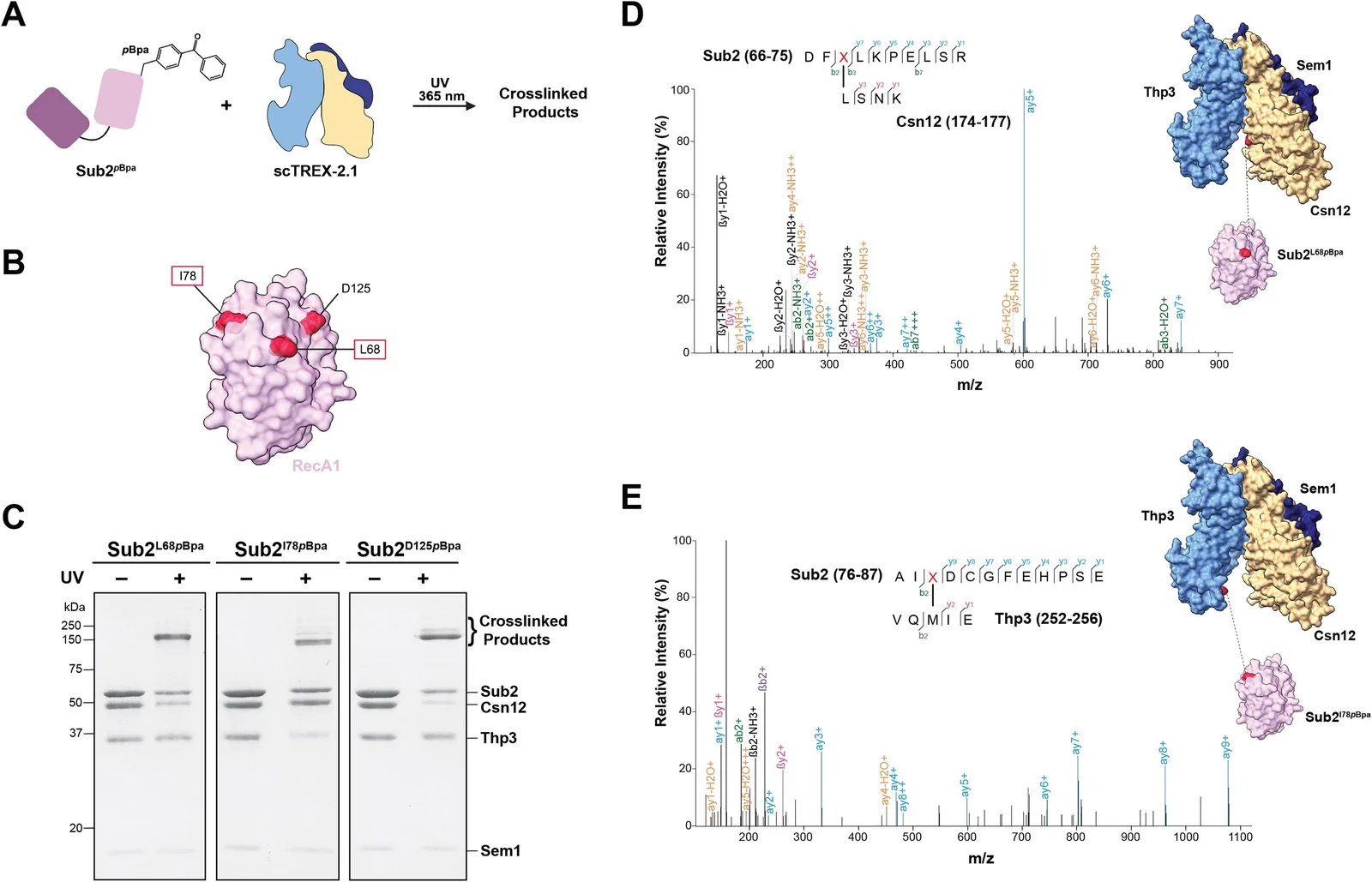
Probing scTREX-2.1 interactions with photoactivatable Sub2*^p^*^Bpa^. (**A**) Schematic for generating the photo-crosslinked scTREX-2.1•Sub2 complexes. *p*Bpa was incorporated at specific residues of Sub2. Sub2*^p^*^Bpa^ was mixed with scTREX-2.1 and treated with ultraviolet light at 365 nm to obtain crosslinked products. (**B**) View of Sub2-RecA1 with *p*Bpa incorporation residues (PBD ID 5SUP). The residues that yielded crosslinking sites by mass spectrometry are boxed. (**C**) SDS–PAGE showing the photo-crosslinking of scTREX-2.1 with Sub2^L68^*^p^*^Bpa^, Sub2^I78^*^p^*^Bpa^, or Sub2^L125^*^p^*^Bpa^, respectively. (**D**) A representative MS/MS spectrum of a Sub2^L68^*^p^*^Bpa^ peptide crosslinked to L174 of the Csn12 subunit of scTREX-2.1. (**E**) A representative MS/MS spectrum of a Sub2^I78^*^p^*^Bpa^ peptide crosslinked to M254 of the Thp3 subunit of scTREX-2.1. X represents *p*Bpa.

To better understand how scTREX-2.1 recognizes Sub2, we sought to map the binding interface using crosslinking mass spectrometry. Photo-crosslinking with the genetically encoded unnatural amino acid (*p*Bpa) allows site-specific capture of protein–protein interfaces upon UV irradiation, and it has been successfully applied in our recent studies [50, 51, 75, 76]. Here, we incorporated *p*Bpa on the surface of Sub2 to photo-crosslink Sub2 with scTREX-2.1 (Fig. 3A). We screened *p*Bpa-labeled Sub2 residues based on their proximity to scTREX-2 in the cryo-EM structure of the scTREX-2•Sub2 complex [50]. The *p*Bpa-incorporated Sub2 proteins were mixed with scTREX-2.1 and treated with UV light at 365 nm. We found that three of the tested residues, Sub2^L68^*^p^*^Bpa^, Sub2^I78^*^p^*^Bpa^, and Sub2^D125^*^p^*^Bpa^, yielded robust crosslinking to scTREX-2.1, as seen by the formation of higher molecular weight products (Fig. 3B and C). In the Sub2^L68^*^p^*^Bpa^ sample, interprotein crosslinks were predominantly mapped to the Csn12 subunit, with the major crosslinking site located at L174 of Csn12 (Fig. 3D and Supplementary Table S5). In contrast, crosslinks from the Sub2^I78^*^p^*^Bpa^ sample were detected exclusively on the Thp3 subunit, with M254 identified as the primary crosslinking site (Fig. 3E and Supplementary Table S6). Together, these results using photo-crosslinking with Sub2*^p^*^Bpa^ probes show that the RecA1 domain of Sub2 contacts both Csn12 and Thp3 in the center of the “V” face of the scTREX-2.1 complex, suggesting a binding mode similar to that in TREX-2.

### Cryo-EM structure of the scTREX-2.1•Sub2 complex

**Figure 4.**
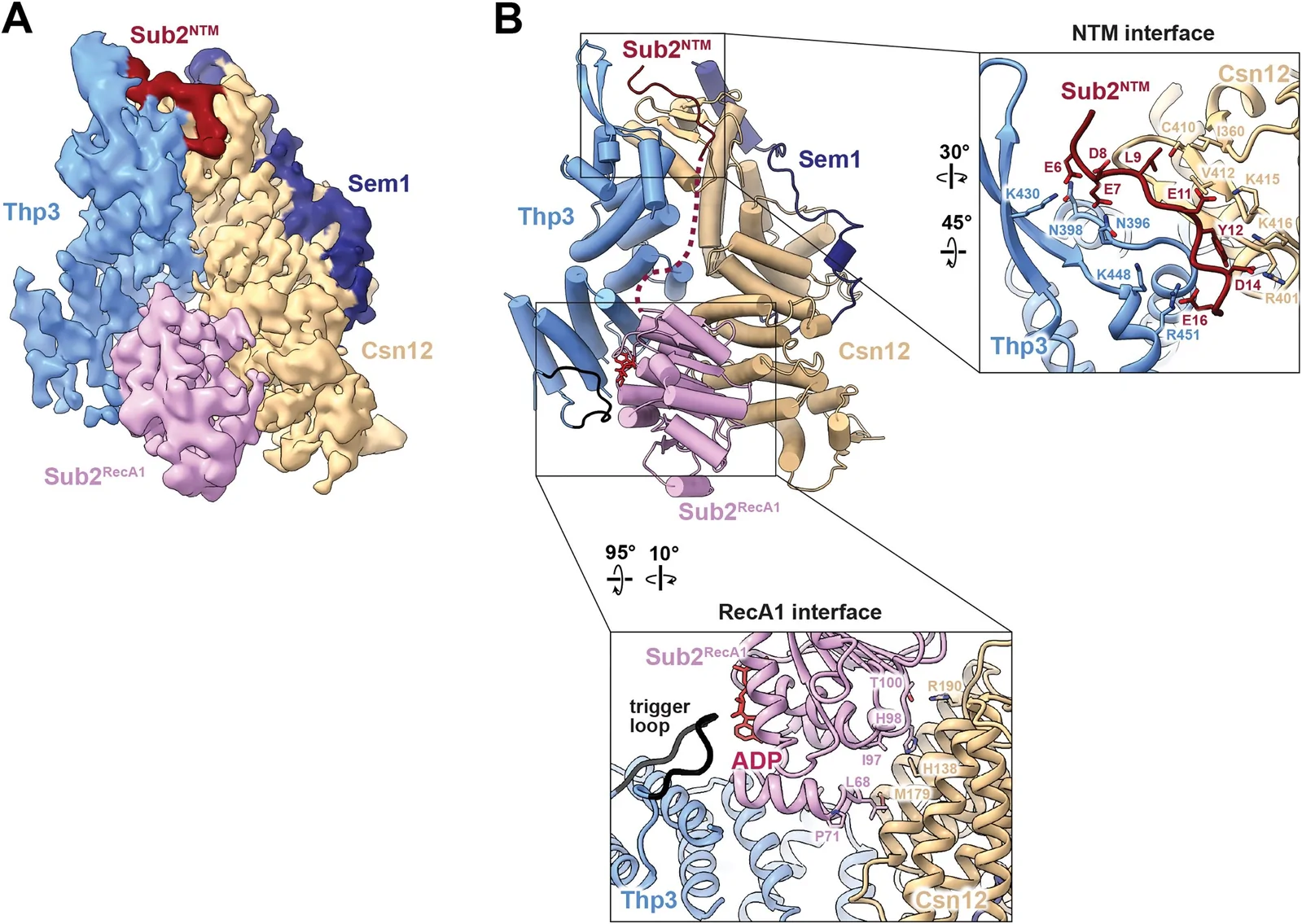
Cryo-EM structure of the scTREX-2.1•Sub2 complex. (**A**) Cryo-EM map of the scTREX-2.1•Sub2 complex in the presence of ADP determined at 3.7 Å resolution. The Thp3, Csn12, and Sem1 subunits of scTREX-2.1 are colored in blue, yellow, and dark blue, respectively. The NTM and the RecA1 domain of Sub2 are colored in red and pink, respectively. (**B**) Structure of the scTREX-2.1•Sub2 complex. Sub2 is observed in a post-hydrolysis state bound to ADP. The scTREX-2.1 interface with the NTM and the RecA1 domain of Sub2 are shown in zoomed-in views.

The three *p*Bpa-incorporated Sub2 variants from the above studies were used to obtain a stabilized scTREX-2.1•Sub2 complex for cryo-EM screening. Our recent biochemical studies showed that the TREX-2 and TREX-2.1 complexes promote the dissociation of Sub2/DDX39B from mRNPs [50, 51]. Mechanistically, our structural studies indicate that the critical trigger loop of TREX-2 or TREX-2.1 engages Sub2/DDX39B in their post-hydrolysis states [50, 51]. Here, we prepared cryo-EM samples in the presence of ADP to mimic a post-hydrolysis state of Sub2. Among the tested samples, the scTREX-2.1•Sub2^D125^*^p^*^Bpa^ complex yielded maps with readily identifiable Sub2 densities and was therefore used for data collection. The cryo-EM data generated a map of the scTREX-2.1•Sub2 complex with an overall resolution of 3.72 Å (Fig. 4A, Supplementary Figs S3–S5, and Supplementary Table S7). The resulting structure shows that the scTREX-2.1 complex binds to the NTM and the RecA domains of Sub2. The *p*Bpa incorporated at residue 125 of Sub2 is crosslinked to the R95 residue of the Csn12 subunit of scTREX-2.1 (Supplementary Fig. S5D), stabilizing the complex at the Sub2-RecA1 interface. While the RecA2 domain of Sub2 can be seen in 2D class averages (Supplementary Fig. S5A), it is largely disordered and thus not modeled in the structure. The scTREX-2.1•Sub2 structure reveals that Sub2 is in a post-hydrolysis state bound to ADP (Fig. 4B and Supplementary Fig. S5C).

The NTM of Sub2 binds to the Thp3-Csn12 interface formed by their WH domains in the scTREX-2.1 complex. Notably, a beta hairpin in the WH domain of Thp3 extends over Sub2-NTM to clamp it at the Thp3-Csn12 interface. The Sub2-NTM interface (Fig. 4B, right) features salt bridges and hydrogen bonds, involving acidic residues (E6, E7, D8, E11, D14, and E16) of Sub2-NTM and basic and polar residues of scTREX-2.1 (N396, N398, K430, K448, and R451 of Thp3; R401, K415, and K416 of Csn12). Hydrophobic interactions also contribute significantly to Sub2-NTM binding to scTREX-2.1. The Y12 residue of Sub2-NTM is buried at the interface with Csn12. Additionally, residue L9 of Sub2-NTM interfaces with a hydrophobic pocket formed by I360, C410, and V412 on the surface of Csn12. Yeast strains carrying mutations on Csn12 (R401, K415, K416) or Thp3 (K448, R451) at the Sub2-NTM binding interface (Fig. 4B) have previously been shown to exhibit intron retention in *IMD4* pre-mRNAs to a similar extent as *csn12Δ* or *thp3Δ* strains [62]. These residues were also shown to contribute to nucleic acid binding of scTREX-2.1 *in vitro* [62]. Based on our structure, the observed intron retention may result from the disruption of Sub2 interactions with scTREX-2.1 and/or nucleic acid interactions.

The scTREX-2.1•Sub2 structure further shows that the NTM of Sub2/DDX39B engages scTREX-2.1 in a manner similar to TREX-2 with two key features: the conserved tyrosine residue (Y12 in Sub2, Y13 in DDX39B) is buried at the interface with TREX-2 and TREX-2.1, mediating a critical hydrophobic interaction, and the highly acidic NTM interacts with the basic surface formed by the two large subunits of TREX-2 or TREX-2.1 [50, 51]. A unique feature of scTREX-2.1 is that Thp3 contains an extended beta hairpin that arches over the other subunit, Csn12, together forming a clamp around the N-terminus of Sub2-NTM. Of note, the Sub2-NTM has been shown to be required for normal growth and mRNA nuclear export, with deletion of residues 6–17 causing a ∼10-fold decrease in growth rate [77]. Additionally, mutation of the conserved Y12 residue or the acidic residues produces a similar growth phenotype [77], suggesting the growth defect in each of these mutant strains may result from disruptions in Sub2 interactions with either or both scTREX-2 and scTREX-2.1.

The RecA1 domain of Sub2 is sandwiched between the helical domains of Thp3 and Csn12 (Fig. 4B, bottom), in line with our crosslinking studies (Fig. 3). On the Thp3 side, an extended loop, which we refer to as “trigger loop,” contacts the ADP binding site of RecA1. On the Csn12 side, several Sub2 loops opposite to the ADP binding site interface with the helical domain of Csn12. Key interactions involve L68, P71, I97, H98, and T100 of Sub2. Comparison of the scTREX-2.1•Sub2 structure with the crystal structure of scTREX-2.1 (Supplementary Fig. S5E) indicates significant flexibility of the helical domain of Thp3 at the interface with Sub2-RecA1. In this case, the helical domain of Thp3 swings toward Sub2-RecA1 in the scTREX-2.1•Sub2 complex, likely caused by their interactions.

### A conserved trigger loop of scTREX-2.1 is critical for Sub2 regulation

**Figure 5.**
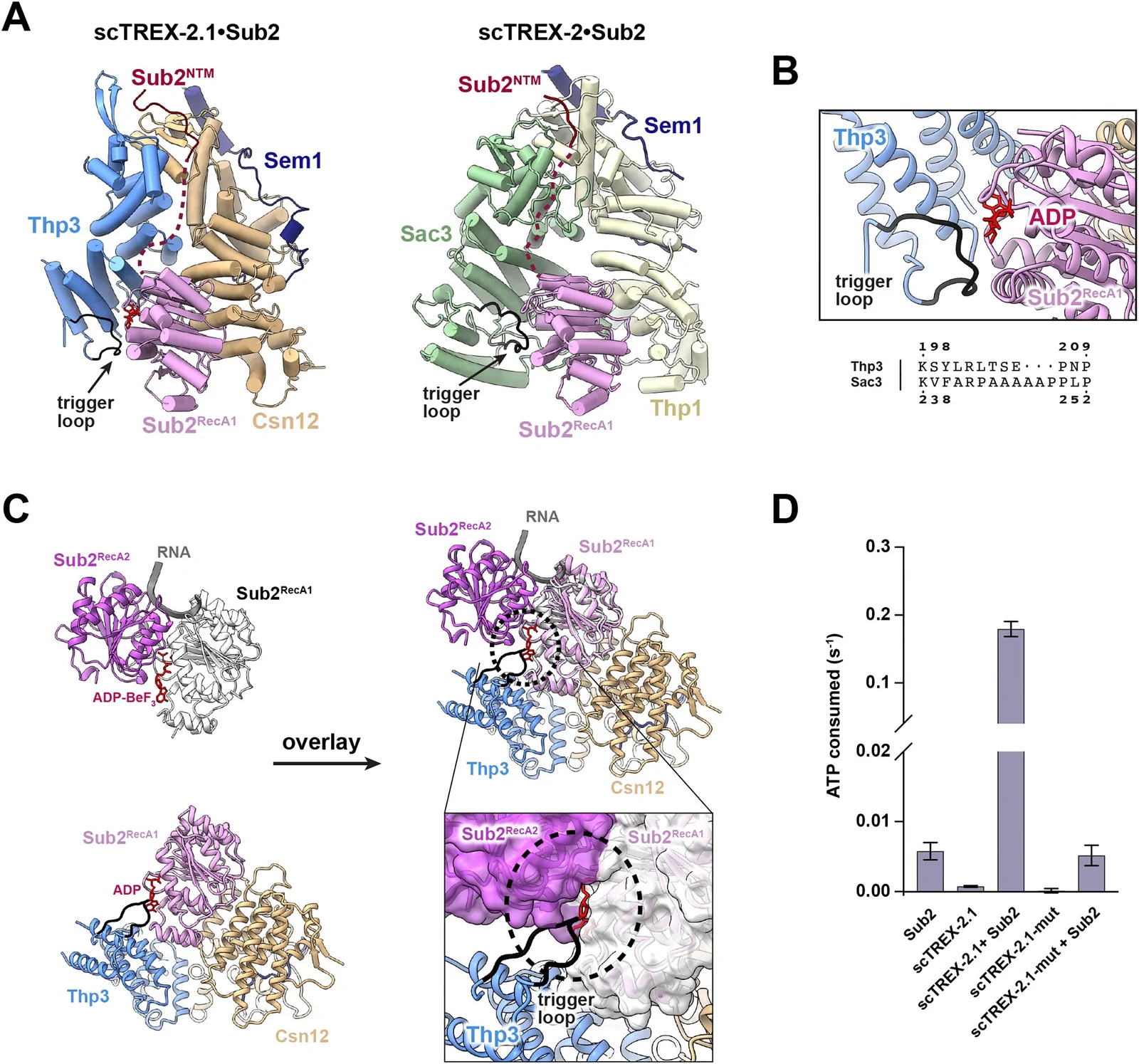
A conserved “trigger loop” is critical for Sub2 regulation by scTREX-2.1. (**A**) Comparison of the structures of scTREX-2.1•Sub2 and TREX-2•Sub2. (**B**) Zoomed-in view of the trigger loop of the scTREX-2.1 complex at the interface with the RecA1 domain of Sub2. The sequence alignment below compares the trigger loop of scTREX-2.1 (on Thp3) and scTREX-2 (on Sac3). (**C**) Overlay of the scTREX-2.1•Sub2 cryo-EM structure and the Sub2•RNA structure (PDB ID 5SUP) by aligning the RecA1 domain of Sub2. A zoomed-in view of the trigger loop shows potential clashes with the RecA2 domain of Sub2 in the context of the Sub2•RNA complex. (**D**) scTREX-2.1 stimulates the ATPase activity of Sub2, and mutation of the trigger loop (scTREX-2.1-mut) diminishes scTREX-2.1′s stimulative effect. In scTREX-2.1-mut, Thp3 ^202^RLTSEP^207^ was replaced with a GGGG linker. Error bars represent the standard deviation of three independent experiments.

Structural comparison of the scTREX-2.1•Sub2 structure and our recently reported TREX-2•Sub2 structure (in the absence of nucleotide) reveals that the Thp3 and Csn12 subunits of scTREX-2.1 are functionally equivalent to the Sac3 and Thp1 subunits of the scTREX-2 complex (Fig. 5A). It is observed that the helical domain of Thp3 is significantly shorter than that of Sac3 and the extended region in Sac3 forms additional interactions with Sub2-RecA1 near the trigger loop [50]. Nevertheless, both scTREX-2.1 and scTREX-2 bind Sub2 in a similar manner, and their respective Thp3 and Sac3 subunits harbor the trigger loop that engages with Sub2-RecA1, which we previously showed is critical for scTREX-2 regulation of Sub2 [50]. Sequence alignment of the trigger loop shows conserved residues KxY/FxR at the N-terminus and enriched proline residues at the C-terminus. In the scTREX-2.1•Sub2 structure, ADP is present and is contacted by the trigger loop (Fig. 5B).

Sub2 undergoes conformational changes during its functional cycle, particularly regarding the relative orientation of the two RecA domains, which is critical for both nucleotide and RNA binding. We aligned the structures of scTREX-2.1•Sub2 with the RNA-bound Sub2, in which the two RecA domains are positioned in a “closed” conformation (Fig. 5C). The alignment shows that the trigger loop of scTREX-2.1 would cause steric clashes with the RecA2 domain of Sub2 if a Sub2•RNA complex is engaged with scTREX-2.1. This observation suggests that the trigger loop may promote separation of the RecA domains and release of Sub2 from RNA, a mechanism resembling that of TREX-2 and TREX-2.1 [50, 51]. To assess the importance of the trigger loop, we generated a mutant scTREX-2.1 (scTREX-2.1-mut), in which ^202^RLTSEP^207^ was replaced with a GGGG linker. The wild-type scTREX-2.1 complex, like the TREX-2 complex, greatly stimulates the ATPase activity of Sub2 (Fig. 5D). In contrast, scTREX-2.1-mut did not stimulate Sub2 (Fig. 5D). Together, our results demonstrate that scTREX-2.1 regulates the activity of Sub2, and the trigger loop of scTREX-2.1 is critical for Sub2 regulation.

**Figure 6.**
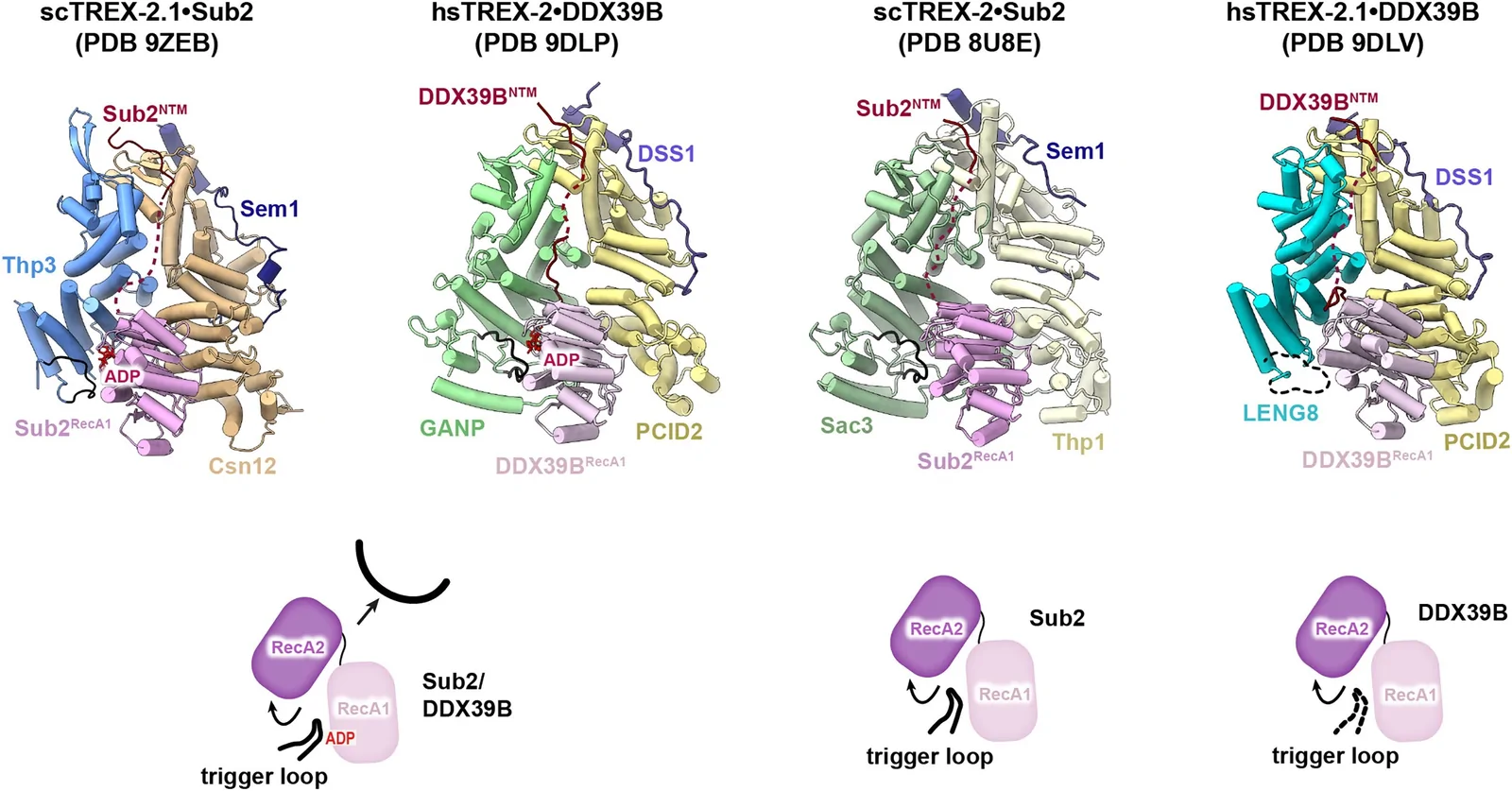
Conserved mechanism of Sub2/DDX39B regulation by TREX-2 and TREX-2.1 complexes from yeast to humans. Cryo-EM structures of the yeast TREX-2.1•Sub2 complex (PDB ID 9ZEB) and the human TREX-2•DDX39B complex (PDB ID 9DLP) represent a post-hydrolysis state in which ADP is bound. Cryo-EM structures of the yeast TREX-2•Sub2 complex (PDB ID 8U8E) and the human TREX-2.1•DDX39B complex (PDB ID 9DLV) represent a post-hydrolysis state in which both RNA and ADP have been released.

Furthermore, our scTREX-2.1•Sub2 complex structure captures a post-hydrolysis state of Sub2 in which RNA has been released while ADP is still present (Fig. 6). Such a conformation was also captured in our recently determined human TREX-2•DDX39B complex structure (Fig. 6) [51]. In comparison, cryo-EM structures of scTREX-2•Sub2 and human TREX-2.1•DDX39B capture Sub2/DDX39B in a state after ADP and RNA have been released (Fig. 6) [50, 51]. In the scTREX-2•Sub2 complex, the trigger loop from Sac3 still engages the RecA1 domain of Sub2. In the TREX-2.1•DDX39B complex, the trigger loop from LENG8 is dislodged. These structures reveal critical snapshots during Sub2/DDX39B regulation by TREX-2 and TREX-2 like complexes. Together, these data indicate that TREX-2 and TREX-2.1 function in a conserved manner from yeast to humans.

### Degradation of the scTREX-2.1 component Csn12 causes the accumulation of intron-containing pre-mRNA

**Figure 7.**
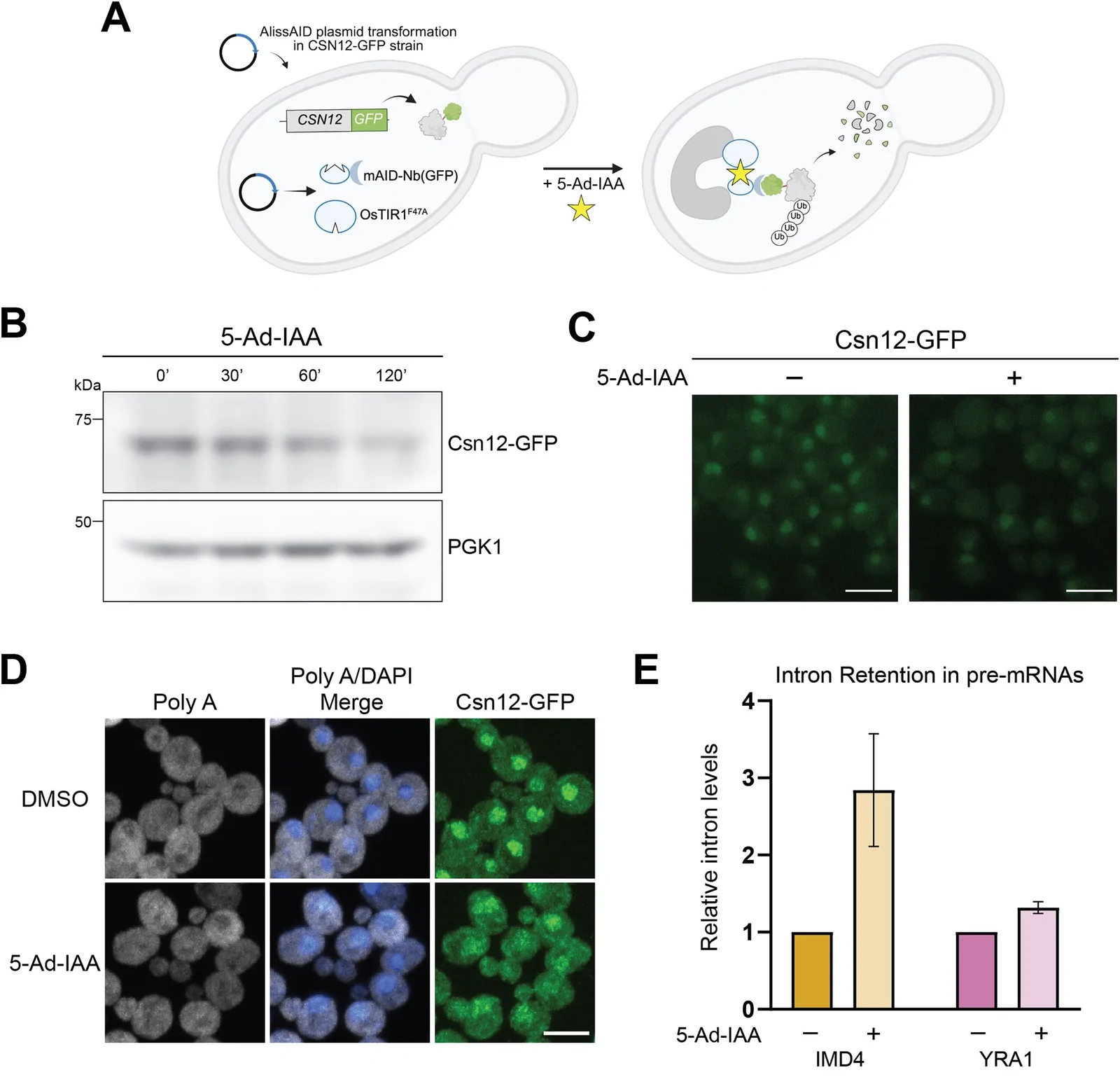
Degradation of scTREX-2.1 component Csn12 by AlissAID system showed accumulation of intron-containing transcripts. (**A**) Schematic for degradation of Csn12 in yeast using the AlissAID system. The CSN12-GFP yeast strain was transformed with an AlissAID plasmid encoding for an AID-tagged GFP nanobody and OsTIR1^F47A^. The addition of 5-Ad-IAA triggers degradation of the GFP-tagged Csn12. (**B**) Degradation of Csn12-GFP detected by western blot after 0-, 30-, 60-, or 120-min treatment of 5-Ad-IAA. (**C**) Representative fluorescent microscopy images of the CSN12-GFP AlissAID strain yeast before and after treatment with 5-Ad-IAA for 2 h. Scale bar, 10 µm. (**D**) Poly(A) RNA FISH in Csn12-GFP depletion condition. Cells were treated by DMSO (control) or 5-Ad-IAA for 2 h. Scale bar, 5 µm. (**E**) Degradation of the Csn12 subunit of scTREX-2.1 causes intron retention. Intron levels of yeast IMD4 and YRA1 were measured before and after treatment with 5-Ad-IAA for 2 h via qPCR. Error bars represent the standard deviation of three biological replicates.

Our biochemical and structural studies indicate that scTREX-2.1, like human TREX-2.1 and TREX-2 complexes, regulates the activity of Sub2/DDX39B. scTREX-2 is enriched at the NPC and likely regulates Sub2 in a spatially regulated manner to facilitate the final steps of mRNP remodeling and their docking and translocation through the NPC. scTREX-2.1 complex, like the human TREX-2.1 complex, is localized inside the nucleus, and its function has remained largely elusive. One observation in yeast *csn12* or *thp3* deletion strains is the accumulation of pre-mRNA for many intron-containing genes [60]. To further understand the role of the scTREX-2.1 complex, and to avoid potential long-term cellular effects that may arise from disrupting the scTREX-2.1•Sub2 complex via *csn12* or *thp3* deletions, we employed an inducible degradation system to temporally control protein degradation. Specifically, we chose the affinity linker-based super-sensitive auxin-inducible degron (AlissAID) system that degrades GFP-tagged proteins (Fig. 7A) [78, 79]. Csn12-GFP protein was efficiently degraded within 2 h after adding 5-Ad-IAA, as shown by western blot and GFP fluorescence imaging (Fig. 7B and C).

To examine the involvement of scTREX-2.1 in the regulation of mRNA export, we analyzed poly(A) RNA subcellular distribution by FISH. However, Csn12 depletion did not cause obvious poly(A) RNA nuclear accumulation phenotype (Fig. 7D). We next assessed the effect of Csn12 degradation on the levels of intron-containing pre-mRNA using qPCR. We first measured the pre-mRNA level of *IMD4*, previously reported to be among the most affected in the *csn12* deletion strain [60, 62], and observed that degradation of Csn12 led to an ∼3-fold increase in *IMD4* pre-mRNA (Fig. 7E). This defect is similar to that observed in the *csn12* deletion strain [62]. We next tested the effect of Csn12 degradation on *YRA1* pre-mRNA. Yra1 is a highly enriched component in nuclear mRNPs, mediating nuclear mRNP packaging and nuclear export, which directly interacts with Sub2 [7, 8, 22, 54]. The intron in the *YRA1* gene plays an important role in auto-regulating Yra1 protein expression in yeast [80]. We observed that Csn12 degradation causes a modest increase in *YRA1* pre-mRNAs (Fig. 7E). Together, acute degradation of the scTREX-2.1 component Csn12 led to an accumulation of intron-containing pre-mRNAs, and the extent of this effect appears to vary among different transcripts. The human TREX-2.1 complex was recently shown to associate with spliceosome components [63, 66]. These findings collectively support conserved roles for TREX-2.1 in splicing and related nuclear mRNP biogenesis pathways.

## Discussion

The DEAD-box protein Sub2, a core component of the TREX complex, is a key mRNP remodeling ATPase in nuclear mRNP assembly, processing, and export. Here, we identify the TREX-2-like complex Thp3-Csn12-Sem1, or scTREX-2.1, as a previously unknown regulator of Sub2. We demonstrate that the scTREX-2.1 complex directly interacts with and stimulates the activity of Sub2 and co-occupies mRNPs formed *in vivo* with Sub2.

### Regulation of the Sub2 ATPase cycle in gene expression

**Figure 8.**
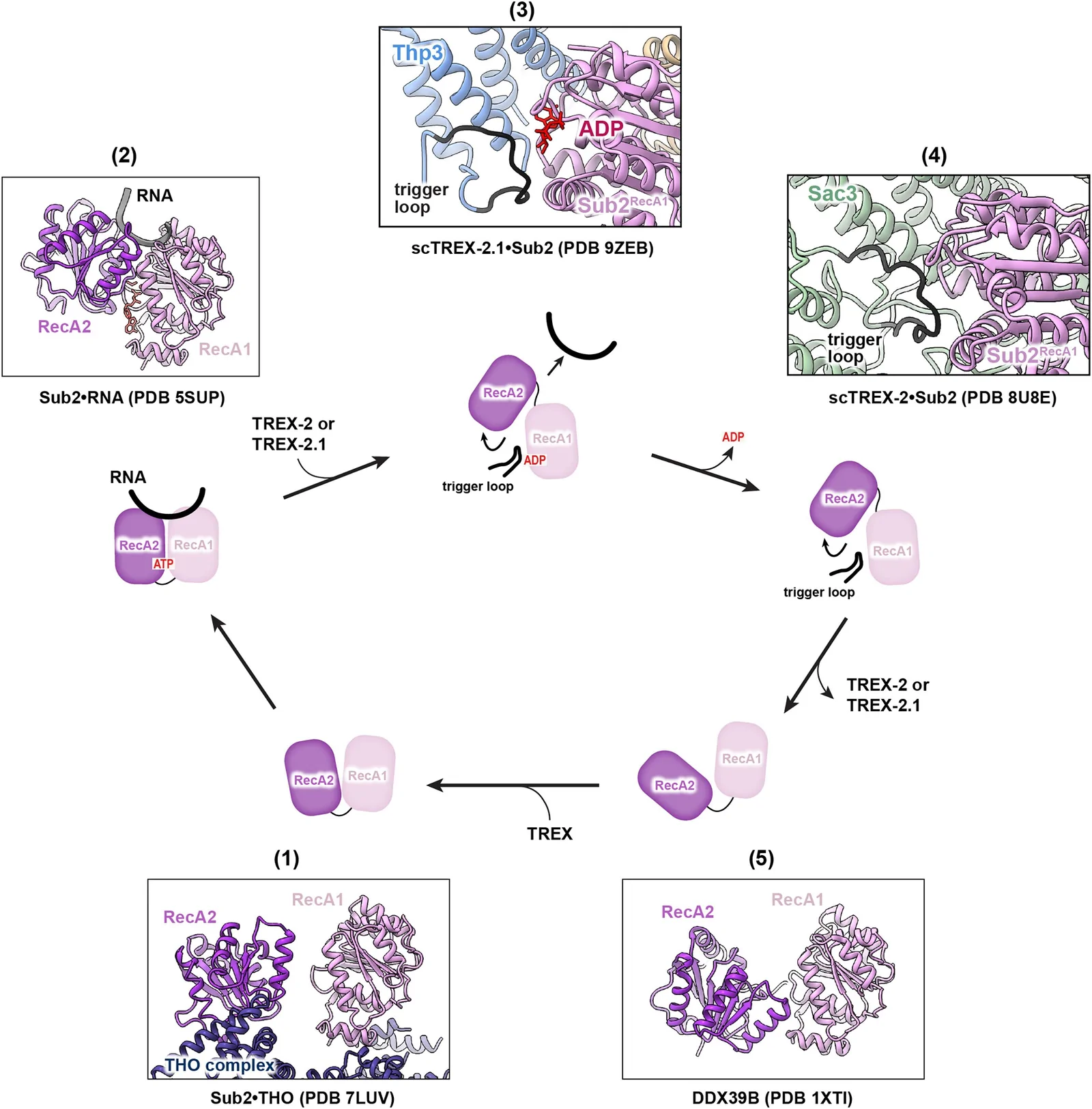
Sub2 ATPase cycle in mRNP remodeling. The THO complex stabilizes a semi-open conformation of Sub2 (PDB ID 7LUV) and primes its engagement with nascent transcripts (state 1). The ATPase forms mRNPs together with export factors such as Yra1 and Tho1 (PDB ID 5SUP and 8ENK) (state 2), facilitating mRNP packaging. scTREX-2 or scTREX-2.1 facilitates the release of Sub2 from the mRNP at distinct steps in nuclear mRNP remodeling, in the nucleus and at the NPC respectively. The structure of the scTREX-2.1•Sub2 complex in this work captures a key post-hydrolysis state in which RNA has been released, yet ADP is still bound to Sub2 (state 3). The scTREX-2•Sub2 structure represents another post-hydrolysis state (PDB ID 8U8E) in which both RNA and ADP have been released from Sub2 (state 4). Sub2 is then recycled for the next round of events (PDB ID 1XTI) (state 5).

The cryo-EM structure of the scTREX-2.1•Sub2 complex adds to our full understanding of Sub2 regulation (Fig. 8). Sub2 contains an unstructured NTM and two RecA domains. The two RecA domains adopt distinct conformations throughout the enzymatic cycle of Sub2. During nuclear mRNP assembly, the THO complex binds Sub2 and positions the two RecA domains in a half-open conformation to facilitate RNA binding (Fig. 8, state 1). Sub2 adopts a closed conformation upon binding to ATP and single-stranded RNA, in which the two RecA domains clamp on the RNA backbone (Fig. 8, state 2). Sub2 can form complexes on RNA with other mRNP assembly factors, like Yra1 and Tho1. scTREX-2 or scTREX-2.1 stimulates the ATPase activity and the release of Sub2 from the RNA, with our scTREX-2.1•Sub2 structure providing a snapshot of the post-hydrolysis state of Sub2 with ADP still present (Fig. 8, state 3). This state highlights that the trigger loop of Thp3 is near ADP, suggesting it may insert itself between the two RecA domains of Sub2 and facilitate Sub2 release from the mRNP. Our previously determined scTREX-2•Sub2 structure captures Sub2 in a state after it has released both ADP and RNA (Fig. 8, state 4). Finally, Sub2 is released from scTREX-2 or scTREX-2.1. Free Sub2 adopts an open conformation, with the two RecA domains moving independently relative to each other, available to engage in the next round of mRNP remodeling (Fig. 8, state 5). These structures provide insights into each step of the functional cycle of Sub2, as well as reveal a conserved molecular mechanism of Sub2 regulation by scTREX-2 and scTREX-2.1 (Figs 6, 8). Critical for the regulation of Sub2 is the trigger loop of the Thp3 or Sac3 of the scTREX-2.1 or scTREX-2 complex, respectively. Mutation of the trigger loop greatly diminished the ability of scTREX-2.1 or scTREX-2 to stimulate Sub2 [50]. This mechanism extends to the TREX-2 complex and the TREX-2.1 complex in humans, as shown in our recent studies [51]. Together, these observations reveal a general mechanism of TREX-2 and TREX-2.1 complexes in regulating the mRNP remodeling ATPase Sub2/DDX39B from yeast to humans.

In the context of gene expression, Sub2/DDX39B’s ATPase cycle governs its mRNP remodeling activity, which likely induce changes in RNA interactions/folding and/or promote protein association/dissociation from the mRNP depending on the specific context. Potential targets of Sub2/DDX39B activity include the TREX complex component Yra1/ALYREF and mRNA export adaptors such as Tho1/SARNP, CHTOP, LUZP4, and UIF [36, 54, 81–83], of which Yra1/ALYREF and Tho1/SARNP have been shown to play critical roles in mRNP packaging for mRNA export [7, 8, 36]. The THO complex, TREX-2, and TREX-2.1 likely facilitate Sub2/DDX39B mediated mRNP remodeling at distinct stages from transcription to export [50–58, 66–68]. Recent studies on the human NPC suggests that human TREX-2 serves as an integral NPC module [84], where TREX-2 can interact with the mRNA export receptor NXF1•NXT1 through its N-terminal nucleoporin homology domain [41]. Evidence further suggests that a fraction of NXF1 resides at the pore to engage mRNP docking at the pore and facilitate transport through the NPC [85]. Therefore, via our work and that of others, it is envisioned that Sub2/DDX39B acts across nuclear mRNP biogenesis to facilitate key mRNP maturation events that drive the mRNP toward an export competent state or nuclear decay.

In the context of scTREX-2.1, how Sub2 functions to influence gene expression and mRNP remodeling is not known. A plethora of evidence has established a role for Sub2/DDX39B in splicing, including its direct interaction with the splicing factor Mud2/U2AF65 [86–92]. DDX39B has also been shown to directly engage the U1 snRNP [93]. In addition, LENG8 of human TREX-2.1 was shown to be recruited to pre-mRNAs by splicing factors, including the U1 snRNP [63, 66]. Using mRNP-SiMPull, we show that Thp3, and thus the scTREX-2.1 complex, resides on mRNPs. Our co-localization analysis further showed co-occupancy of Thp3 and Sub2 in ∼10%–15% of mRNPs, as well as a significant population with each factor present independent of the other (Fig. 2). This suggests that scTREX-2.1 may be involved in Sub2 regulation on a subset of target mRNPs. In yeast, intron-containing genes are limited but highly expressed, thus, it is in line with ∼15% of scTREX-2.1 co-occupancy, which may be related to Sub2 and scTREX-2.1 engaging in the context of splicing/spliced mRNPs. Notably, deletion of *thp3* or *csn12* causes a transcription defect of intronless genes [61], suggesting intronless genes may also be the target of scTREX-2.1.

### The role of TREX, TREX-2, and TREX-2.1 complexes

Nuclear mRNP biogenesis is a highly integrated process that links transcription, pre-mRNA processing, quality control, and nuclear export. Accordingly, the functions of TREX, TREX-2, and TREX-2.1 span multiple stages of this pathway. In addition to their potential role in splicing, both scTREX and scTREX-2.1 have been implicated in transcription. TREX and its associated factors, such as Gbp2 and CTDK-1, as well as scTREX-2.1, are recruited to actively transcribed genes [17, 61, 94–96]. Deletion of various scTREX components impairs transcription and results in transcription-dependent hyperrecombination [17]. Notably, scTREX-2.1 mutants, *thp3Δ* and *csn12Δ*, suppress defects of the THO mutant *hpr1Δ*, highlighting the functional link between scTREX-2.1 and scTREX [61]. Recent studies suggest that yeast and human TREX-2.1 mediates an evolutionarily conserved spliceosome-exosome pathway in nuclear mRNA surveillance [65]. In yeast, scTREX-2.1 associates with early splicing factors such as Mud2, which is known to interact with Sub2, and exosome components including Rrp6 and Rrp47 [65]. Human TREX-2.1 was shown to be recruited to pre-mRNAs by splicing factors including U1 snRNP and linked to RNA quality control through association with the PAXT complex that recruits the nuclear exosome to polyadenylated RNAs [65–67]. Notably, in yeast, the scTREX-associated SR-like proteins Gbp2 and Hrb1 also have established roles in nuclear mRNA quality control [55, 95, 97]. In addition to quality control, recent studies on human TREX-2.1 have shown that TREX-2.1 affects the nucleocytoplasmic ratio of a subset of mRNAs and modulates alternative mRNA processing [51, 68].

TREX and its associated factors further play important roles in mRNP packaging [7, 8]. Yra1/ALYREF engages nascent transcripts by recognizing the CBC and facilitates mRNP assembly and export [9, 29, 30, 33, 54]. Our recent work showed that deletion of Yra1 results in decreased compaction of nuclear mRNPs in yeast [8]. Tho1/SARNP (MOS11 in *Arabidopsis*) is another Sub2/DDX39B binding partner that was identified as a high-copy suppressor of the THO mutant *hpr1Δ* in yeast [98–102]. Sub2/DDX39B uses a partially overlapping interface to interact with THO or Tho1/SARNP [36], suggesting that Tho1 may bind Sub2 after THO dissociates from Sub2 on the mRNP. Alternatively, THO and Tho1 may function in parallel pathways during mRNP assembly [98]. Yra1 and Tho1 can promote higher-order Sub2 complexes through multivalent interactions [36]. Like the scTREX-2 complex [50], the interaction between scTREX-2.1 and Sub2 appears to be compatible with Tho1 and Yra1 (Supplementary Fig. S6). Our studies suggest that both TREX-2.1 and TREX-2 are capable of remodeling mRNP complexes containing Sub2/DDX39B, Yra1/ALYREF, and Tho1/SARNP. While recent studies suggest scTREX-2 regulates Sub2 at the NPC, the cellular context of Sub2 regulation by scTREX-2.1 remains an important question. The highly integrated nature of nuclear mRNP biogenesis makes it challenging to dissect the contributions of individual factors at specific steps. The structural and functional findings presented here provide a molecular framework for understanding how scTREX-2.1 regulates Sub2 and will facilitate future studies investigating the coordinated functions of TREX, TREX-2, and TREX-2.1 in nuclear mRNP biogenesis.

## Supplementary Material

gkag884_Supplemental_File

## Data Availability

The cryo-EM maps generated in this study have been deposited in EMDB under accession codes EMD-74098. The coordinates have been deposited in PDB under the DOI https://doi.org/10.2210/pdb9zeb/pdb. The mass spectrometry data have been deposited to the ProteomeXchange Consortium via the PRIDE partner repository with the dataset identifier PXD073414.
