# Supplementary material for: Conserved mRNP remodeling mechanism of the TREX-2.1 complex
Source: Nucleic Acids Res. 2026 Sep 12;54(17):gkag884. doi: 10.1093/nar/gkag884 (PMC13569498; doi:10.1093/nar/gkag884)
Supplement: Supplementary file 1 [file gkag884_supplemental_file.pdf]

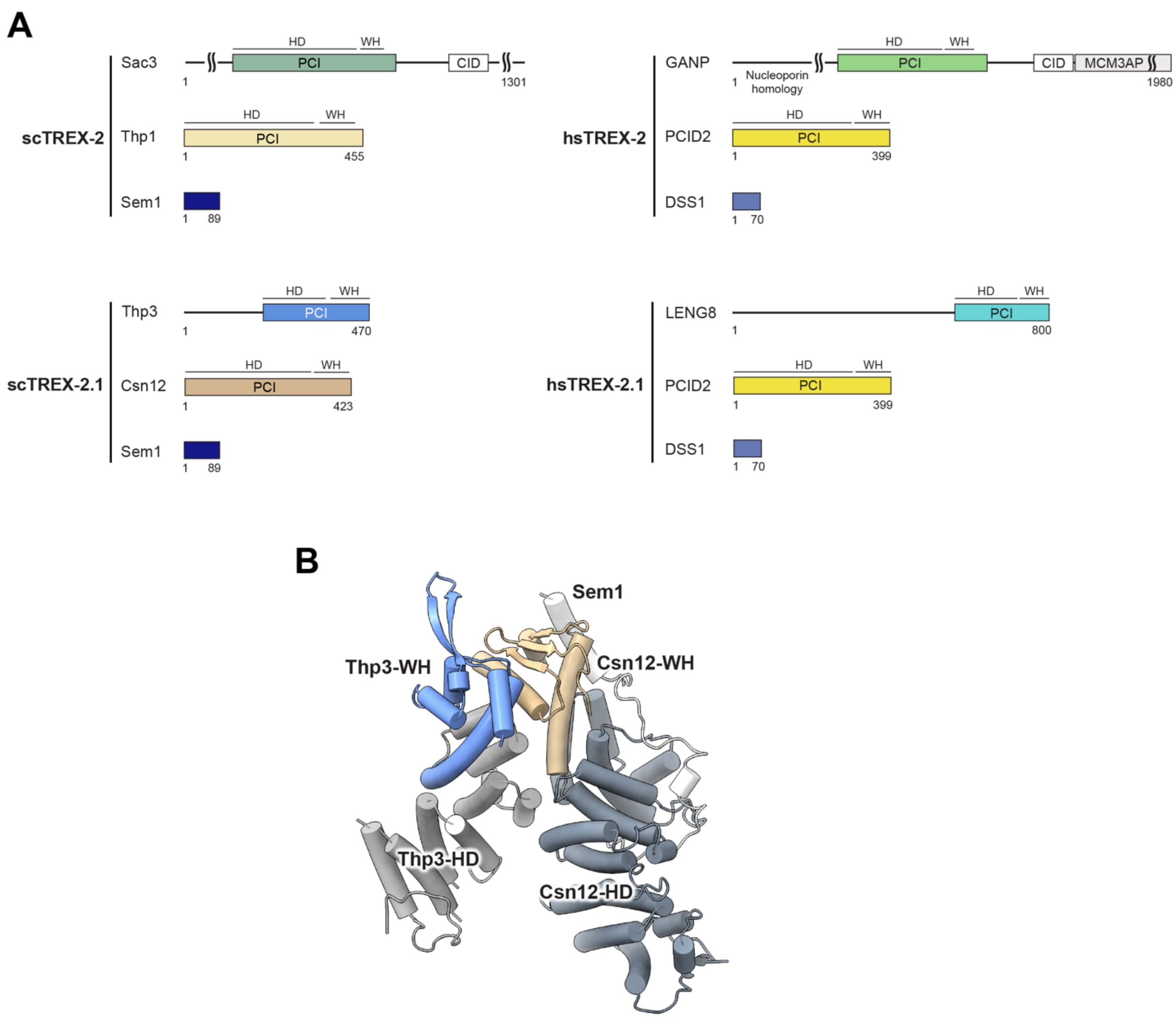

Figure S1. TREX-2 and TREX-2.1 complexes.
(**A**) Schematics of TREX-2 and TREX-2.1 complexes in yeast and humans. (**B**) PCI fold in the yeast TREX-2.1 complex. The winged-helix (WH) and the helical (HD) domains of Thp3 and Csn12 are shown in different colors to illustrate their domain organization.

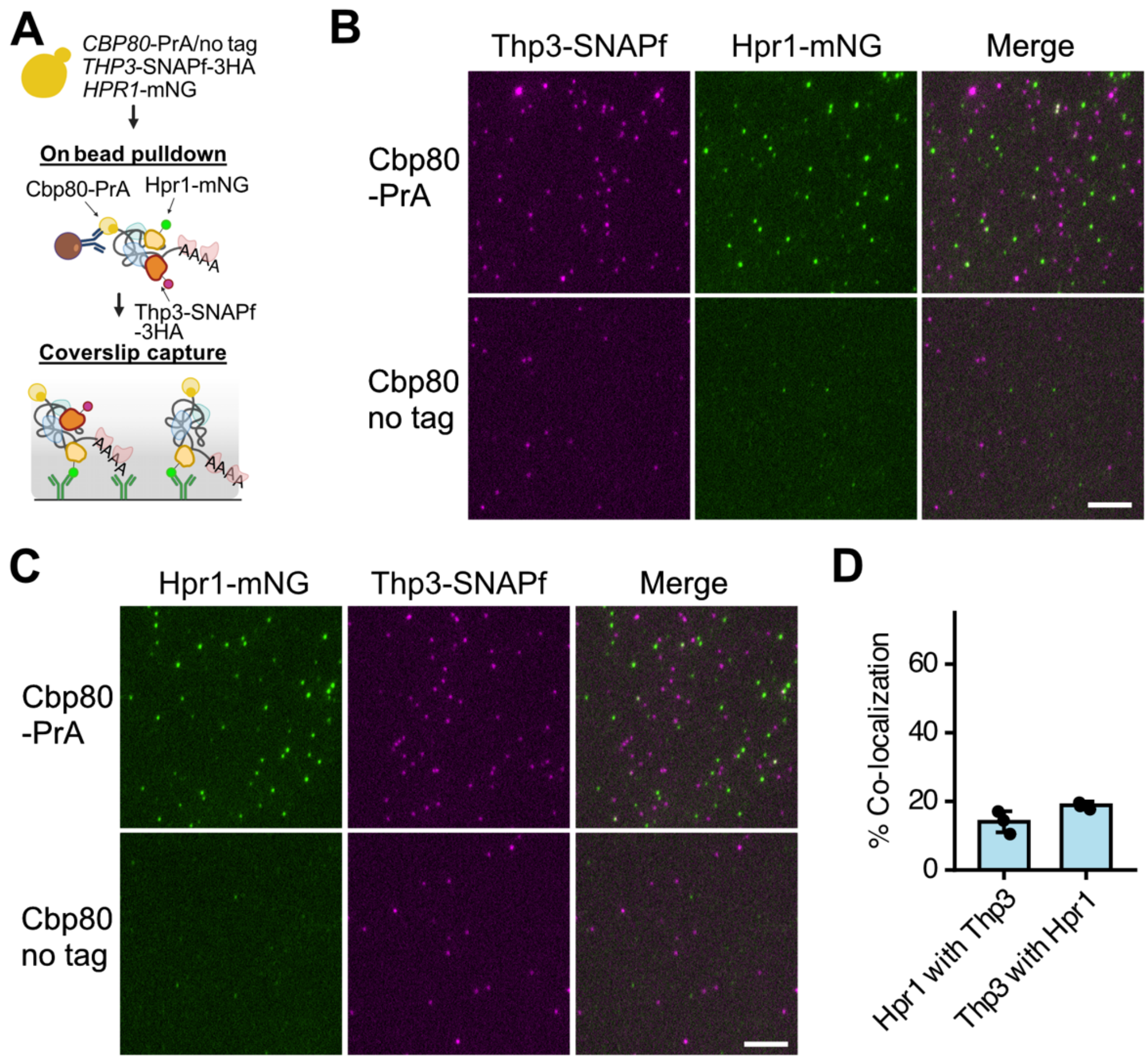

Figure S2. scTREX-2.1 and THO complex can co-occupy CBC-bound mRNPs
(**A**) Cartoon depicts mRNP isolation procedure to capture and determine co-localization of Thp3-SNAPf-3HA and Hpr1-mNG in CBC-bound mRNPs by TIRF microscopy. (**B and C**) Representative TIRF images of Thp3-SNAPf and Hpr1-mNG. Isolated CBC-bound mRNPs were captured on the glass surface via anti-HA for the analysis of Hpr1 co-localization with Thp3 (panel B) or anti-mNG antibody for the analysis of Thp3 co-localization with Hpr1 (panel C). Scale bar, 5 µm. (**D**) Graph shows percent co-localization frequency with error bars displaying standard deviation and replicate data as black dots.

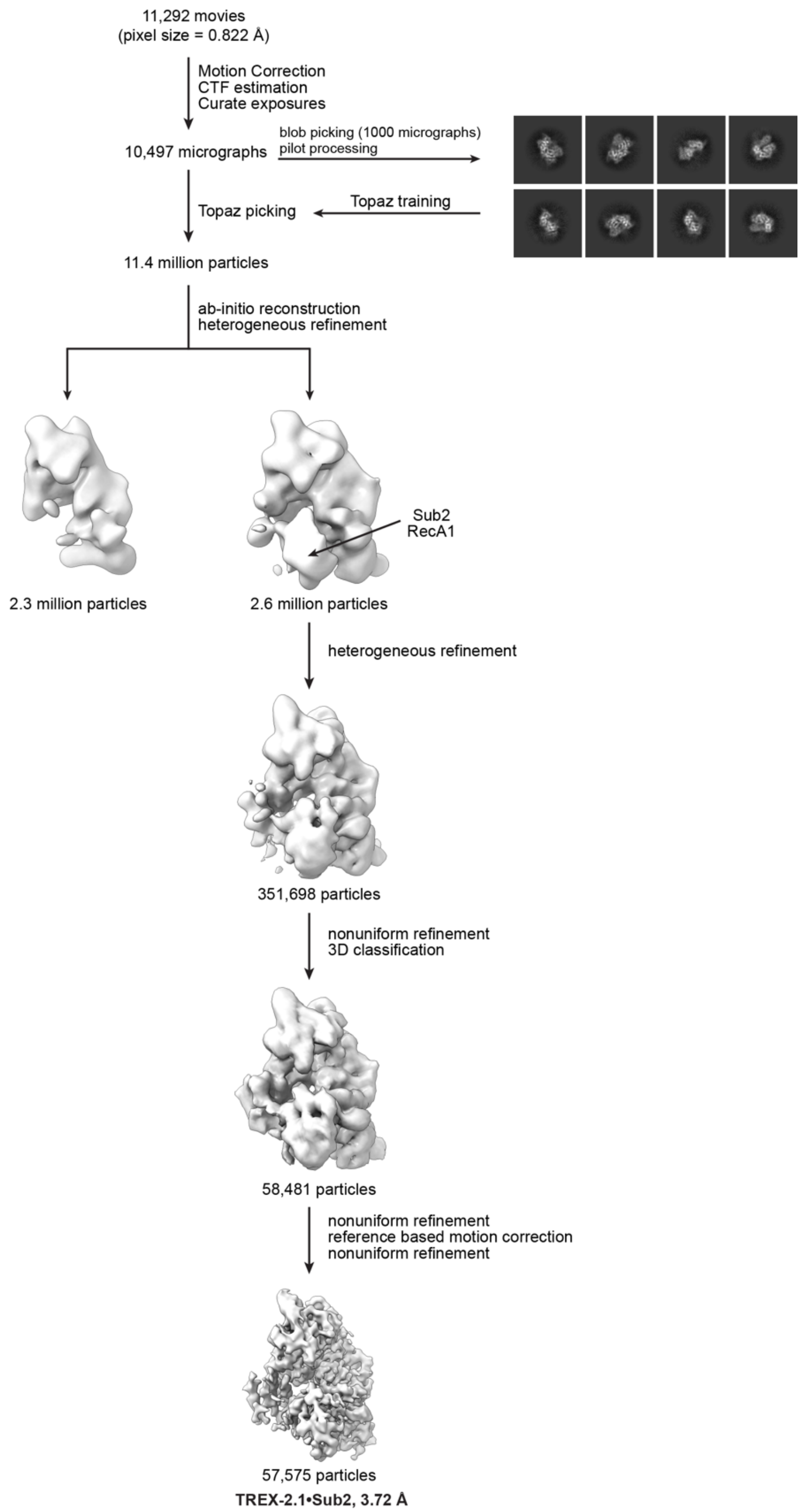

Figure S3. Cryo-EM data processing workflow for the scTREX-2.1•Sub2 complex. A total of 11,292 movies were collected on a 300 keV Titan Krios cryo-electron microscope. Data were processed in CryoSPARC. A set of 57,575 particles yielded a reconstruction of a scTREX-2.1•Sub2 map at 3.72 Å resolution.

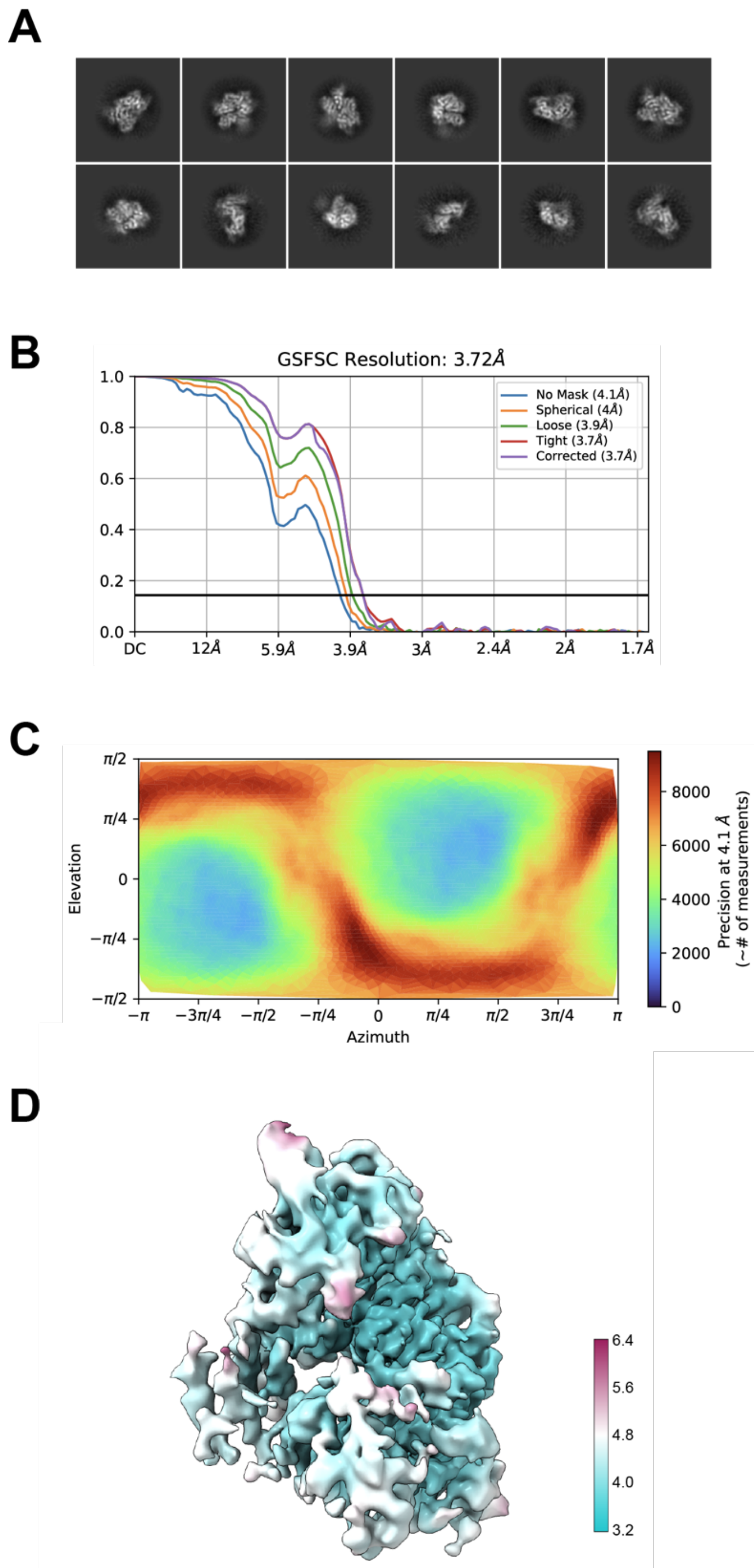

Figure S4. Cryo-EM reconstruction of the scTREX-2.1•Sub2 complex.
(**A**) Selective 2D class averages from the final particle set of scTREX-2.1•Sub2. (**B**) The gold-standard Fourier shell correlation (GSFSC) curves of the final reconstruction of scTREX-2.1•Sub2. (**C**) Angular distribution of the final reconstruction of scTREX-2.1•Sub2 computed in CryoSPARC. (**D**) Local resolution of the scTREX-2.1•Sub2 cryo-EM map.

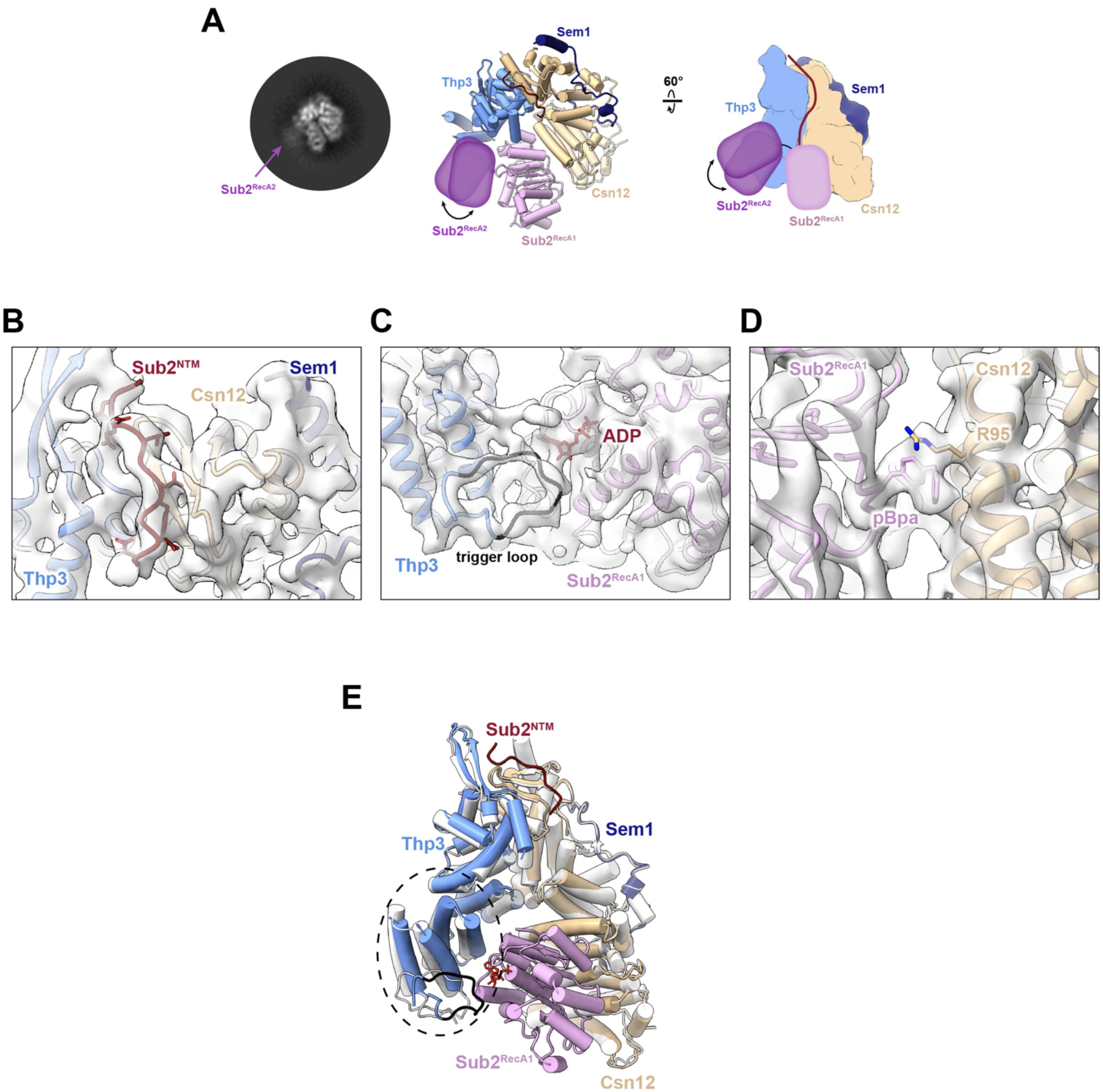

Figure S5. Structure model of the scTREX-2.1•Sub2 complex.
(**A**) The RecA2 domain of Sub2 is mobile in the scTREX-2.1•Sub2 complex. Left: a 2D class average from the final data set with putative RecA2 density as indicated. Middle: scTREX-2.1•Sub2 complex orientated in a similar angle. Right: cartoon of the scTREX-2.1•Sub2 complex. (**B**) Cryo-EM density map at the scTREX-2.1 interface with the NTM of Sub2. (**C**) Cryo-EM density map at the interface of the Thp3 trigger loop and the RecA1 domain of Sub2. (**D**) Cryo-EM density map at the *p*Bpa crosslinking site. The *p*Bpa125 of Sub2 is crosslinked to Arg95 of the Csn12 subunit. (**E**) Comparison of the scTREX-2.1•Sub2 cryo-EM structure and the scTREX-2.1 crystal structure (PDB ID 7EWM, colored in gray). The Thp3 region harboring the trigger loop, as indicated by the circle, undergoes a conformational change upon binding to Sub2.

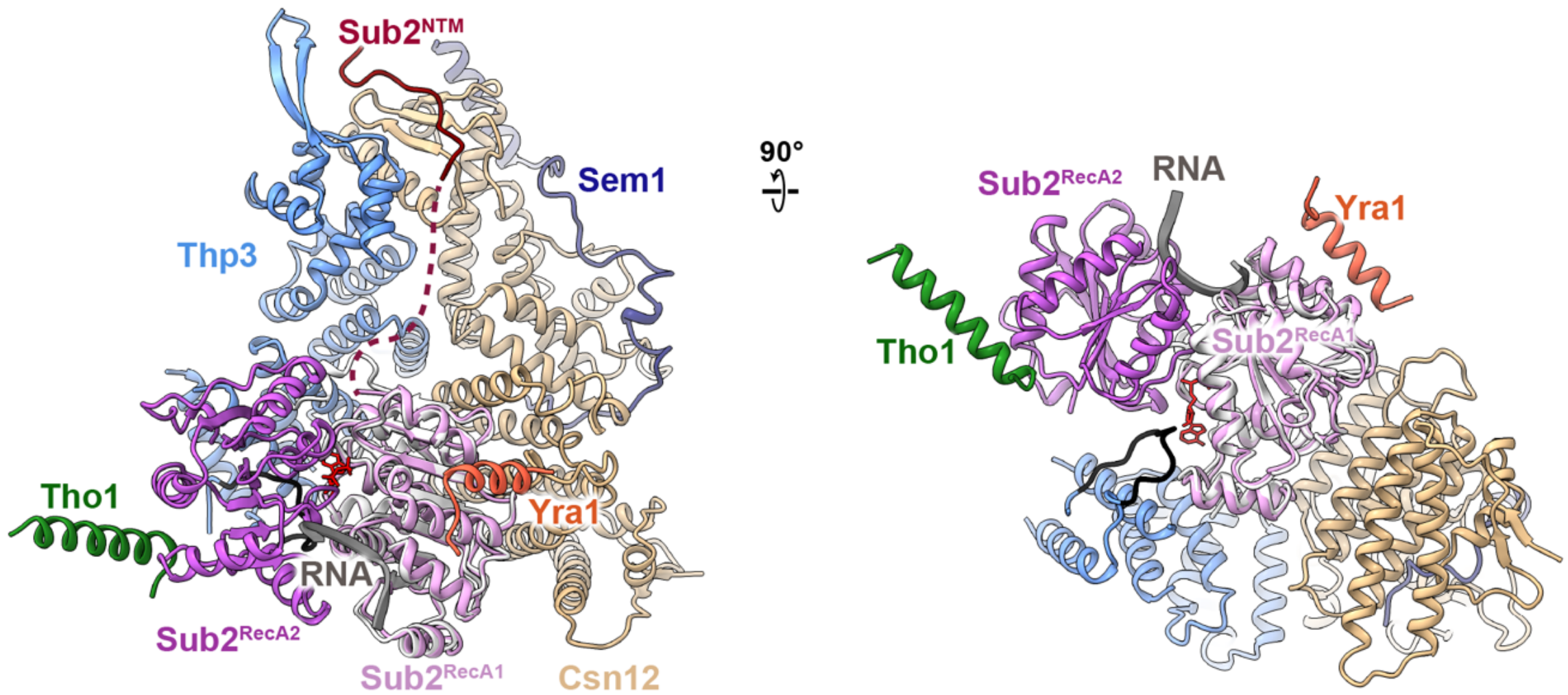

Figure S6. TREX-2.1 interaction with Sub2 is compatible with Yra1 and Tho1.
The scTREX-2.1•Sub2 structure is overlaid with the Sub2•Yra1•RNA structure (PDB ID 5SUP) by aligning the RecA1 domain of Sub2. Similarly, the scTREX-2.1•Sub2 structure is overlaid with the DDX39B•Tho1•RNA structure (PDB ID 8ENK); for clarity, only Tho1 is shown, and DDX39B and RNA are omitted. The scTREX-2.1•Sub2 complex and the Sub2•RNA complex are colored as in Fig. 5C. Yra1 is colored in orange-red, and Tho1 is colored in green.

Table S1. List of reagents used in this study

Reagent	Company	Catalog #
**Chemicals**		
*p*Bpa	AEchem	AE021642
5-Ad-IAA	Tokyo Chemical Industry	A3390
TRIzol™ Reagent	Invitrogen	15596026
Concanavalin-A	Thermo Fischer	J61221.MC
poly(A) RNA	Cytiva	GE27-4110-01
ATP	Sigma	A7699
Phosphoenolpyruvate	Sigma	860077
NADH	Sigma	N8129
Pyruvate Kinase/Lactate Dehydrogenase	Sigma	P0294
PLL(20)-g[3.5]-PEG(2)/PEG(3.4)-biotin 20%	SuSoS	PLL(20)-g[3.5]-PEG(2)/PEG(3.4)-biotin 20%
Streptavidin	Prozyme	SA10
BSA	Sigma	C3400
Casein	Sigma	A4503
Pluronic F-127	Sigma	P2443
κ-casein	Sigma	C0406
RNase inhibitor murine	NEB	M0314L
Antiform B	Sigma	A5757
SNAP-Surface 649	NEB	S9159S
Dynabeads M270 Epoxy	Invitrogen	14302D
Rabbit IgG	Sigma	I5006
HRV3C protease	AG Scientific	H-1192
Catalase	Sigma	C-40
Glucose oxidase	Sigma	G2133
Trolox	Sigma	238813
TetraSpeck	Invitrogen	T7279

Vanadyl Ribonucleoside Complex	NEB	S140S
Zymolyase 20T	US Biological	Z1000
E.coli tRNA	Sigma	10109541001
DABCO (1,4-diazabicyclo-2.2.2 octane)	Fisher	AC112470250
DAPI	Sigma	D9542
Custom LNA Oligonucleotide (Cy-labeled dT probe)	Qiagen	339998-P2-QTE-35357-C
**Antibodies**		
Anti-GFP	Sigma	11814460001
Anti-PGK1	Abcam	ab113687
Anti-mouse IgG	Cell Signaling	7076
Anti-mouse-biotin	Invitrogen	31800
Anti-mNG	Chromotek	32F6
Anti-HA	Sigma	H3663
Anti-V5	Invitrogen	R960-25
**Kits**		
Fast SYBR™Green Master Mix	Thermo Fischer	4385612
SuperScript III First-Strand Synthesis System	Invitrogen	18080-051
RQ1 RNase-free DNase	Promega	M6101
**Specialized Equipment (ATPase Assays)**		
BioTek Synergy HTX microplate reader		
**Specialized Equipment (qPCR Assays)**		
QuantStudio 3 Real-Time PCR system		

Table S2. List of plasmids used in this study

Plasmid	Description	Origin
BYP10392	pAlissAID anti-GFP_316 (*URA3* marker)	Ogawa et. al., 2023
pEVOL-pBpF	Addgene plasmid #31190	Chin et al., 2002
pGST-TEV-Thp3(140-470)		This study
pGST-TEV-Thp3(140-470)-mut	Thp3 trigger loop mutant: ^{202}RLTSEP^{207} replaced with GGGG linker	This study
pRSFDuet-Csn12(1-423)-Sem1(1-89)	His-tagged (TEV cleavable) Csn12 and untagged Sem1	This study
pGST-TEV-Sub2(1-446)		Xie et. al., 2025
pGST-TEV-Sub2(1-446)-bpa68	amber stop codon TAG incorporated at residue 68 of Sub2	This study
pGST-TEV-Sub2(1-446)-bpa78	amber stop codon TAG incorporated at residue 78 of Sub2	This study
pGST-TEV-Sub2(1-446)-bpa125	amber stop codon TAG incorporated at residue 125) of Sub2	Xie et. al., 2025

Table S3. List of oligos used in this study

Oligo #	Sequence	Description
PAA37	TCAACGTCGAAGGTTTGCCA	Forward primer for qPCR of *YRA1* intron
PAA38	TTGATCAAAAGCGTGTGCCA	Reverse primer for qPCR of *YRA1* intron
PAA39	AGGAACAGGCTTCCATGGTC	Forward primer for qPCR of *IMD4* intron
PAA40	AAGCGCATTCTAATGAAAGAAACA	Reverse primer for qPCR of *IMD4* intron

Table S4. Yeast Strains

Strain #	Genotype
BMY4419	*MATa, his3Δ1, leu2Δ0, lys2Δ0, met15Δ0, ura3Δ0, THP3-mNG::HIS3MX*
BMY4441	*MATa, his3Δ1, leu2Δ0, lys2Δ0, met15Δ0, ura3Δ0, THP3-mNG::HIS3MX, CBP80-PPX-PrA::NatMX*
BMY5696	*MATa, his3Δ1, leu2Δ0, lys2Δ0, met15Δ0, ura3Δ0, THP3-SNAPf-3HA::hphMX, CBP20-3V5-TEV-Biotin-His::URA3MX*
BMY5698	*MATa, his3Δ1, leu2Δ0, lys2Δ0, met15Δ0, ura3Δ0, THP3-SNAPf-3HA::hphMX, CBP20-3V5-TEV-Biotin-His::URA3MX, CBP80-PPX-PrA::NatMX*
BMY5851	*MATa, his3Δ1, leu2Δ0, lys2Δ0, met15Δ0, ura3Δ0, HPR1-SNAPf-3HA::hphMX, SUB2-mNG::HIS3MX, CBP20-3V5-TEV-Biotin-His::URA3MX*
BMY5853	*MATa, his3Δ1, leu2Δ0, lys2Δ0, met15Δ0, ura3Δ0, HPR1-SNAPf-3HA::hphMX, SUB2-mNG::HIS3MX, CBP20-3V5-TEV-Biotin-His::URA3MX, CBP80-PPX-PrA::NatMX*

BMY5694	*MATa, his3Δ1, leu2Δ0, lys2Δ0, met15Δ0, ura3Δ0, THP3-SNAPf-3HA::hphMX, HPR1-mNG::HIS3MX,*
BMY5700	*MATa, his3Δ1, leu2Δ0, lys2Δ0, met15Δ0, ura3Δ0, THP3-SNAPf-3HA::hphMX, HPR1-mNG::HIS3MX, CBP80-PPX-PrA::NatMX*
BCY46	*MAT*a, *his3∆1, leu2∆0, met15∆0, ura3∆0, CSN12-GFP::HIS3*
AAY8	*MAT*a, *his3∆1, leu2∆0, met15∆0, ura3∆0, CSN12-GFP::HIS3, [pAlissAID anti-GFP_316]*

Table S5. Analysis of Sub2$^{L68p\mathrm{Bpa}}$ crosslinks to scTREX-2.1

Sub2$^{L68p\mathrm{BPa}}$ peptide[a]	Csn12 peptide	Site of *p*Bpa crosslink[b]	Spectral Count
^{66}[DFXLKPELSR]^{75}	^{152}[LNENK]^{156}	L152 (2)	2
^{66}[DFXLK]^{70}	^{174}[LSNK]^{177}	L174 (1)	1
^{66}[DFXLKPELSR]^{75}	^{174}[LSNK]^{177}	L174 (32)	32
^{66}[DFXLKPELSR]^{75}	^{174}[LSNKDMIK]^{181}	L174 (27)	27
^{66}[DFXLKPELSR]^{75}	^{178}[DMIK]^{181}	D178 (16)	16
^{66}[DFXLKPELSR]^{75}	^{195}[DIPPLNK]^{201}	D195 (17), P197 (23)	40
**Sub2$^{L68p\mathrm{BPa}}$ peptide[a]**	**Thp3 peptide**	**Site of *p*Bpa crosslink[b]**	**Spectral Count**
^{66}[DFXLKPELSR]^{75}	^{199}[SYLR]^{202}	S199 (3)	3

[a]X=*p*Bpa
[b]Number in parentheses indicates the spectral count for the indicated crosslink site.

Table S6. Analysis of Sub2$^{I78p\mathrm{Bpa}}$ crosslinks to scTREX-2.1

Sub2$^{I78p\mathrm{BPa}}$ peptide[a]	Thp3 peptide	Site of *p*Bpa crosslink[b]	Spectral Count
^{76}[AIXD]^{79}	^{203}[LTSEPNPDLIRPPNILQK]^{220}	P207 (2)	2
^{76}[AIXD]^{79}	^{252}[VQMIE]^{256}	V252 (2), M254 (9), I255 (2)	13
^{76}[AIXD]^{79}	^{252}[VQMIENSFTIK]^{262}	M254 (1)	1
^{76}[AIXDCGFEHPSE]^{87}	^{252}[VQMIE]^{256}	M254 (4), I255 (1)	5

[a]X=*p*Bpa
[b]Number in parentheses indicates the spectral count for the indicated crosslink site.

Table S7. Cryo-EM data collection, processing, and refinement statistics of the scTREX-2.1•Sub2 complex

	**scTREX-2.1•Sub2** **(PDB 9ZEB; EMD-74098)**
**Data collection and processing**	
Microscope	Krios G4
Voltage (kV)	300
Camera	Gatan K3
Electron exposure (e^-/Å^2)	57.3
Defocus range (μm)	-0.8 to -2.0
Pixel size (Å)	0.822
Initial particle images (no.)	11,427,446
Final particle images (no.)	57,575
Symmetry imposed	C1
Box size (pixels)	288
Map resolution (masked, Å)	3.72
FSC threshold	0.143
**Refinement**	
Initial model used	PDB 7EWM and 5SUP
Model resolution (Å)	4.1
FSC threshold	0.5
Map sharpening B factor (Å^2)	60
Model-to-map CC (mask)	0.74
Model composition<br>Protein residues<br>Ligand	 946<br>1
B factors (Å^2)<br>Protein<br>Ligand	 175<br>197
R.m.s. deviations<br>Bond lengths (Å)<br>Bond angles (°)	 0.003<br>0.534
Validation<br>MolProbity score<br>Clashscore<br>Poor rotamers (%)	 1.73<br>9.96<br>0.11
Ramachandran plot<br>Favored (%)<br>Allowed (%)<br>Disallowed (%)	 96.66<br>3.34<br>0
